# Comparative transcriptomics reveal developmental turning points during embryogenesis of a hemimetabolous insect, the damselfly *Ischnura elegans*

**DOI:** 10.1038/s41598-017-13176-8

**Published:** 2017-10-19

**Authors:** Sabrina Simon, Sven Sagasser, Edoardo Saccenti, Mercer R. Brugler, M. Eric Schranz, Heike Hadrys, George Amato, Rob DeSalle

**Affiliations:** 1 Biosystematics Group, Wageningen University & Research, Droevendaalsesteeg 1, 6708 PB Wageningen, The Netherlands; 2 Sackler Institute for Comparative Genomics, American Museum of Natural History, Central Park West and 79th St., New York, NY 10024 USA; 3 Ludwig Institute for Cancer Research, Karolinska Institutet, 17177 Stockholm, Sweden; 4 Laboratory of Systems and Synthetic Biology, Wageningen University & Research, Stippeng 4, 6708 WE Wageningen, The Netherlands; 5 Biological Sciences Department, NYC College of Technology, City University of New York, 300 Jay Street, Brooklyn, New York, 11201 USA; 6 ITZ, Ecology&Evolution, University of Veterinary Medicine Hanover, Buenteweg 17d, D-30559 Hannover, Germany; 7 Yale University, Department of Ecology & Evolutionary Biology, 165 Prospect Street, New Haven, CT 06511 USA

## Abstract

Identifying transcriptional changes during embryogenesis is of crucial importance for unravelling evolutionary, molecular and cellular mechanisms that underpin patterning and morphogenesis. However, comparative studies focusing on early/embryonic stages during insect development are limited to a few taxa. *Drosophila melanogaster* is the paradigm for insect development, whereas comparative transcriptomic studies of embryonic stages of hemimetabolous insects are completely lacking. We reconstructed the first comparative transcriptome covering the daily embryonic developmental progression of the blue-tailed damselfly *Ischnura elegans* (Odonata), an ancient hemimetabolous representative. We identified a “core” set of 6,794 transcripts – shared by all embryonic stages – which are mainly involved in anatomical structure development and cellular nitrogen compound metabolic processes. We further used weighted gene co-expression network analysis to identify transcriptional changes during Odonata embryogenesis. Based on these analyses distinct clusters of transcriptional active sequences could be revealed, indicating that embryos at different development stages have their own transcriptomic profile according to the developmental events and leading to sequential reprogramming of metabolic and developmental genes. Interestingly, a major change in transcriptionally active sequences is correlated with katatrepsis (revolution) during mid-embryogenesis, a 180° rotation of the embryo within the egg and specific to hemimetabolous insects.

## Introduction

During embryogenesis, the central life cycle, the embryonic body plan is laid out, starting with blastoderm formation, germ band formation, followed by elongation, segmentation, and appendage formation. Most of our knowledge about developmental gene networks during insect embryogenesis is built on the *Drosophila* paradigm, which is far from being universal^1^. In addition, the involvement of genes in specific developmental processes is usually determined on a small scale by comparing expression patterns of specific key genes across species by means of *in situ* hybridization or quantitative RT-PCR. This approach has identified genes with deep conservation of expression patterns, that have also been shown to underlie developmental similarities on unexpectedly large evolutionary scales^2,3^. However, given that genes commonly function together, concerted expression changes of distinct sets of genes may often be phenotypically relevant. In this context, transcriptomic developmental time courses have already demonstrated the use of *de novo* assembled transcriptomes spanning various developmental stages to identify developmental genes and members of signalling pathways and to explore genome-level questions^4–6^. However, comparative molecular studies focusing on early/embryonic stages during insect development are limited to a few taxa, mainly holometabolous insects, especially the model systems like the fruitfly (*Drosophila melanogaster*), the red flour beetle (*Tribolium castaneum*), or the parasitic wasp (*Nasonia vitripennis)* ^7–10^. In contrast, a few hemimetabolous insect species, e.g. *Oncopeltus* and *Gryllus*, have become more widely used, but have been investigated only for selected key *Drosophila* homologs^11–16^. Although several studies have extensively examined morphological changes during hemimatabolous embryogenesis^17–19^, large-scale embryonic transcriptomic studies are still missing.

Here, we attempt to fill in this gap and present the first comparative embryonic transcriptome for the blue-tailed damselfly *Ischnura elegans*. *I. elegans* belongs to the family Coenagrionidae of the suborder Zygoptera (damselfly) within the order Odonata. Odonata have become a model organism for studies in ecology and evolutionary biology and currently serves different research aspects like assessing the impact of global warming^20,21^, trait-dependent diversification patterns^22,23^, colour vision^24,25^ and colour polymorphism evolution^26–28^ (for a review see also Bybee *et al*.^29^ and references therein). There is also an increasing source of Odonata molecular studies^30–33^ and recently a study comprising the first draft genome of an Odonata species was published^34^. In addition, Odonata represent a promising system for future evo-devo research. They represent one of the two earliest pterygote (winged) insect orders^35–37^. Consequently, studying the evolution of developmental processes in an Odonata representative would provide crucial insights in key mechanisms underlying the origin and diversification of insect wings.

In the present study, we generated expression data throughout all embryonic developmental stages covering germ band formation, elongation, segmentation, and appendage formation, by performing comprehensive RNA sequencing on single *I. elegans* embryos. Based on this RNA-seq data we developed a novel *I*. *elegans* reference transcriptome and examined gene expression divergence across all embryonic stages to provide novel insights in the genetics of embryogenesis of a hemimetabolous insect. The *de novo* reference transcriptome is undoubtedly valuable for further ecological and evolutionary studies in Odonata. Furthermore, our comparative data will provide insights into the extent of gene expression variation during embryogenesis in more “primitive” hemimetabolous lineages.

## Methods

### Insect Sampling

A mating wheel of *Ischnura elegans* was collected in Southern-France in June 2012. To obtain the egg clutch, the mating wheel was placed in an oviposition chamber that consisted of a vial containing only wet filter paper. After termination of copulation, the male was released and the female was kept overnight in the vial for egg oviposition. The wet filter paper in the vials is known to be sufficient to elicit oviposition in some odonate species^38,39^. On nine subsequent days starting the day after oviposition, approximately 20 eggs of the egg clutch were preserved in RNAlater once at the same time of the day and stored at −80 °C. On the 10^th^ day, no embryos were further preserved as the first nymphs of the egg clutch hatched.

### 454-Squencing Approach

For RNA extraction, several embryos spanning two to three days were pooled together (day 1–3, day 4–5, day 6–7 and day 8–9, Supplementary Table S1). Total RNA extraction and cDNA synthesis was conducted as described in Kvist *et al*.^40^. In total, four cDNA Rapid Libraries (RL) with different indexed barcodes were prepared using a Roche 454 GS RL Prep Kit by following manufacturer’s protocols as outlined in the Roche 454 RL Preparation Method Manual (Roche Applied Sciences, Indianapolis, IN, USA). Emulsion-based clonal amplification (PCR) was carried out using the GS Junior Titanium emPCR (Lib-L) Kit and following manufacturer’s protocols as outlined in the emPCR Amplification Method Manual (Lib-L). This manual was also used for bead recovery, DNA library bead enrichment, and sequence primer annealing. Enriched beads were prepared for sequencing on a GS Junior Titanium PicoTitrePlate Device using the GS Junior Titanium Sequencing Kit and following manufacturer’s protocols as outlined in the Sequencing Method Manual. Massively parallel single-end pyrosequencing was conducted by one multiplexed run on a 454 GS Junior at the Sackler Institute for Comparative Genomics, American Museum of Natural History, New York, NY, USA.

Post-sequencing processing was conducted as described in Kvist *et al*.^40^ followed by trimming of low quality regions; only bases between positions 59–500 and those with a Phred quality score ≥ 25 and a minimum length of 20 base pairs (-v -t 25 -l 20 -Q 33) were retained in the data set using FASTX_trimmer and FASTQ_quality_trimmer (both part of the FASTX toolkit; http://hannonlab.cshl.edu/fastx_toolkit/). Before assembly the raw reads were further checked for potential contamination through local Blast against UniVec (ftp://ftp.ncbi.nlm.nih.gov/pub/UniVec/, accessed Oct 7, 2014) using BLASTN (-reward 1 -penalty −3 -evalue 700 -searchsp 1750000000000 -dust yes -gapopen 3 -gapextend 3). Raw sequences were considered to contain potential contamination if the alignment length of the query with the target exceeded 25 base pairs (bp) and were filtered out using custom perl scripts (VecScreenFilter.pl, compare_2Files.pl, bad_data_uniq.pl; available upon request) and seqtk (https://github.com/lh3/seqtk, accessed Oct 8, 2014). Afterwards, iAssembler tool (v1.3.2.) (-a 10 -b 10 –d) was used to cluster and assembly contigs to obtain unigene sequences^41^. Raw sequence reads can be found in the SRA database under BioProject PRJNA401426.

### Illumina Squencing Approach

Following the Smart-seq 2 protocol^42^, we prepared 15 different Nextera indexed RNA Seq libraries each representing a single embryo and including a replicate of each embryonic developmental stage (expect day 1, 2 and 5; Supplementary Table S1). These developmental stages were defined according to the day after oviposition. Libraries were sequenced on two lanes of 2 × 150bp on a Illumina HiSeq 2500 at the NY Genome Center, New York, NY, USA.

The raw Illumina reads for each of the 15 libraries were delivered as individual fastq files. The Illumina reads were quality-filtered, and sequencing and indexing adapters were removed using Trimmomatic (0.32)^43^ (PE; Final_Adapter-Trim.txt:2:30:10; LEADING:3; TRAILING:3; SLIDINGWINDOW:4:20; MINLEN:35). Only reads with a minimum length of 35 bp were further kept. Overlapping paired-end reads were merged using Flash (1.2.11)^44^ setting max-overlap to 135 bp. Before assembly, the raw reads were further checked for potential contamination through local Blast against UniVec (ftp://ftp.ncbi.nlm.nih.gov/pub/UniVec/, accessed Oct 7, 2014) using search parameters and filtering criteria as described above. Raw sequence reads can be found in the SRA database under BioProject PRJNA401426.

Trinity *in silico* read normalization (trinityrnaseq_r20140413p1)^45^ was applied to remove redundant reads before assembly. Here, the remaining reads of the 15 libraries were normalized together with published Illumina reads from an adult male of *Ischnura elegans* ^46^ using default commands with a max coverage of 50. Orphan reads that resulted due to the trimming and merging step were separately normalized (only left orphans (trimmed R1 reads and merged reads) and right orphans (only R2 orphans)) using the same commands except the paired reads options. *De novo* assembly was conducted using Trinity (trinityrnaseq_r20140413p1)^45^ using default parameters with a minimum kmer coverage of 2 and with the paired modus including the orphans to left and right reads, respectively.

### Building of the Reference Transcriptome for Gene Expression Analyses

Bacterial genomic contamination is common in eukaryotic samples^47^. Therefore, the pre-assemblies were checked for human and bacterial sequence contamination using DeconSeq^48^, with an alignment identity threshold of 97% (−i 97) and an alignment coverage threshold of 90% (-c 90). Both pre-assemblies were analysed separately against the Human Reference (GRCh37; ftp://ftp.ncbi.nih.gov/genomes/Homo_sapiens/ARCHIVE/BUILD.37.2/Assembled_chromosomes/seq/; accessed July 25, 2014), and 5,242 unique bacterial genomes (ftp://ftp.ncbi.nih.gov/genomes/Bacteria/; accessed Jan 28, 2015) with a cross-check (-dbs_retain) against *Drosophila melanogaster* (ftp://ftp.flybase.net/genomes/Drosophila_melanogaster/dmel_r6.01_FB2014_04/fasta/; accessed July 27, 2014) and *Acyrthosiphon pisum* (aphidbase_2.1b_mRNA; https://www.aphidbase.com/aphidbase; accessed July 28, 2014). In addition, in order to reduce the redundancy of the pre-assemblies, they were first processed by CD-HIT-EST (v4.6.1-2012-08-27)^49^ with 95% identity to remove identical fragments.

The resulting contigs of both pre-assemblies (contamination-reduced and assembly improved) were merged using CAP3 (VersionDate: 12/21/07)^50^ to reduce potential redundancy. To improve the overall quality of the hybrid assembly likely coding regions with a minimum open reading frame (ORF) length of 200 bp were extracted from the transcripts using TransDecoder from the Trinity package^45^. The hybrid assembly was used as a reference transcriptome for the weighted gene correlation network analyses (WGCNA). For theses transcripts, the base-level coverage was calculated using bowtie2 (v2.2.5)^51^ and aligning all Illumina reads against the hybrid assembly. To calculate the mean coverage per base genomeCoverageBed of bedtools2^52^ was applied. Transcripts with a mean coverage per base of less then 5 bp were removed from the final reference transcriptome. This Transcriptome Shotgun Assembly project has been deposited at DDBJ/EMBL/GenBankunder the accession GFWX00000000. The version described in this paperis the first version, GFWX01000000. The final reference transcriptome is available in the TSA database under BioProject PRJNA401426. The completeness of the reference transcriptome was assessed using CEGMA (v2.5)^53^ and BUSCO (v1.1b1)^54^. Functional annotation and analysis of the reference transcriptome was conducted using the Trinotate pipeline (v.2.0)^45^. All transcripts and transdecoder-predicted proteins with a minimum length of 200 bp were used as query for BLASTX and BLASTP search, respectively, against the SwissProt non-redundant and the Uniref90 database (both accessed March 2016). Protein domains were predicted using HMMER (v. 3.1b2)^55^ against the Pfam-A database (v.28)^56^, signal peptides were predicted using the SignalP 4.1 server^57^, and transmembrane regions were predicted using the TMHMM server v2.0^58^. RNAMMER (v.1.2)^59^ was used to identify rRNA genes.

### Transcript Quantification and Co-Expression Analyses

Illumina-reads from each embryonic sample were separately aligned to our *de novo* reference transcriptome using bowtie2 (v2.2.5)^51^ and the isoform/gene abundances were estimated using express (v1.5.1)^60^. The resulting count matrix was filtered by abundance based on count-per-million (CPM) values as converted with edgeR (3.8.6)^61^ (R version 3.1.3). Here, differences in library sizes between samples are taken into account and only genes with at least 5 counts in one of the libraries were kept. Following the common approach when constructing gene correlation networks, genes with variance smaller than twice the observed overall variance were also removed since low variance genes represent noise and may hamper the reconstruction of co-expression networks. The resulting filtered count matrix of 27,027 genes was normalized by the trimmed-mean of M values (TMM) method implemented in edgeR and log2 transformed. Using these 27,027 genes, a step-by-step signed hybrid co-expression network was built using WGCNA (v. 1.49) R package^62^. The adjacency matrix was created by calculating the biweight mid-correlation between all genes and by restricting the number of excluded outliers (maxPOutliers = 0.1). These settings have less sensitivity to outliers^63^ as compared to Pearson’s correlation but also takes into account the potential risk of unwanted results when the data have a bi-modal distribution^64^. Outliers are expected due to the high biological heterogeneity in our samples (e.g. long time-span, not an inbred culture, see also Results & Discussion).

Based on the scale-free topology criterion^65^, the power for calculating the adjacency matrix was set to 22 resulting in an R^2^ = 0.86 for the scale-free fit). Genes were hierarchical clustered based on the TOM-based dissimilarity (Topological Overlap Measure (TOM)) and modules (clusters of highly correlated genes) were detected using DynamicTreeCut^66^ with a minimum module size of 50. The resulting 78 identified modules were further merged when their eigengenes (the first principal component of module expression pattern) showed a correlation of 0.9^67^. The correlation coefficients between the resulting 34 merged modules and different ‘traits’ were calculated.

## Results and Discussion

### ***De Novo*** Hybrid Assembly of The Transcriptome of *Ischnura Elegans*

**Figure 1:**
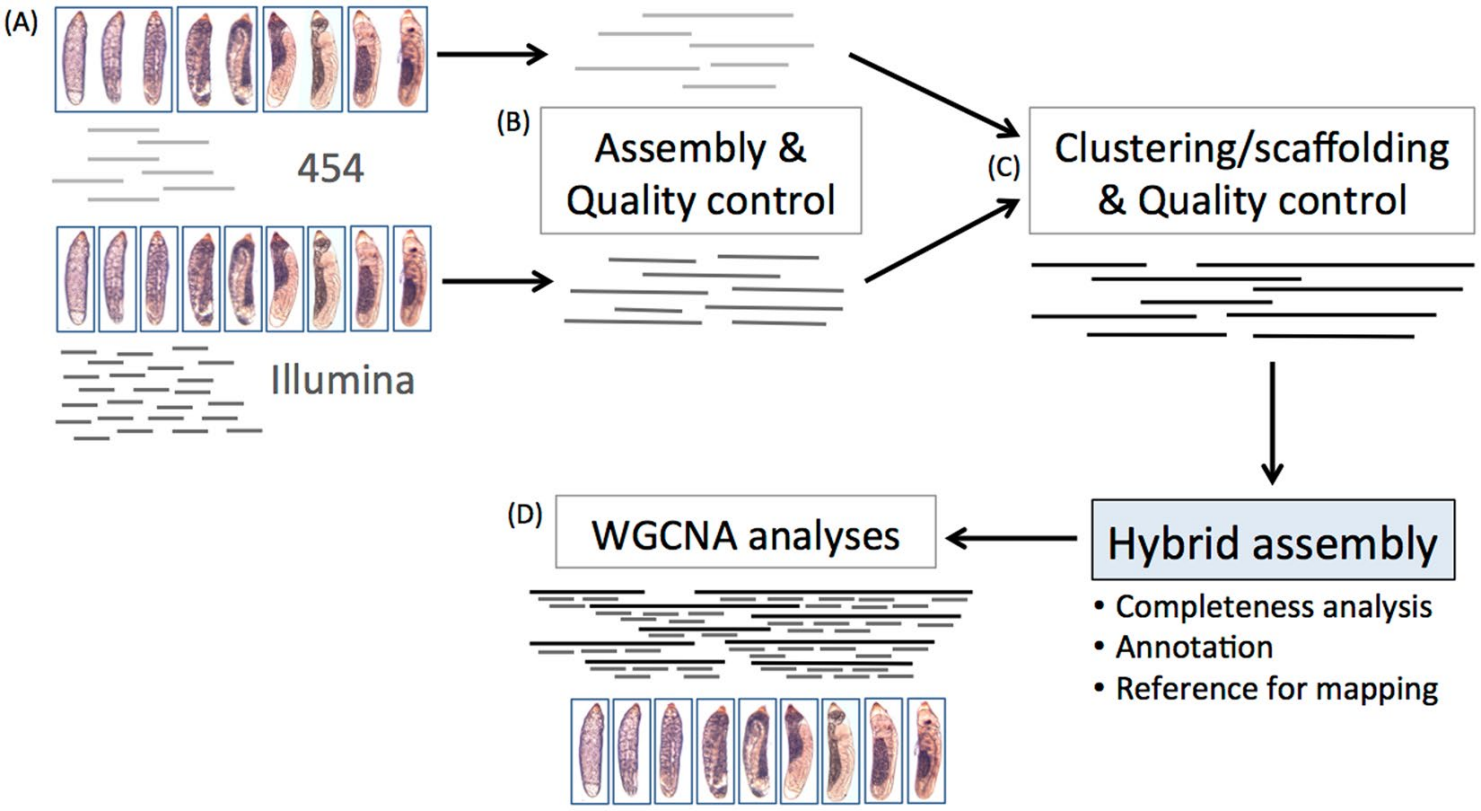
Workflow for the *de novo* hybrid assembly and differential expression (DE) analyses. (**A**) For the 454 sequencing approach several embryos (~20) from several days were pooled together (day 1–3, day 4–5, day 6–7 and day 8–9). For the Illumina sequencing approach one single embryo of each embryonic developmental stage was successfully prepared using the Smart-seq 2 protocol. (**B**) 454 and Illumina reads were separately pre-assembled using iAssembler and Trinity, respectively. Both *de novo* pre-assemblies were separately checked for contamination and redundancy. (**C**) Filtered pre-assemblies were clustered and resulted into the reference hybrid assembly. (**D**) For the WGCNA analyses the Illumina reads from the separate embryonic stages were mapped against the resulting reference hybrid assembly.

To establish the first gene expression profiles during embryonic development of a hemimetabolous insect, two sequencing approaches were conducted for *I. elagans* (454 & Illumina) (see Fig. 1 for an overview of the workflow). To prepare the cDNA for both approaches the same egg clutch were used and all embryonic life stages were included (in total 9 days until nymphs hatched at day 10).

A total of 111,393 454-sequence reads and 149,254,447 Illumina-sequence reads were obtained. The number of raw reads for each library and resulting reads after trimming and cleaning is provided in Supplementary Table S2. The 454 data was *de novo* pre-assembled into 58,271 contigs and (including 3,550 singletons) with a total number of 2,2145,630 bp and a sequence length range from 20 bp to 4,549 bp (Supplementary Table S3). Before assembly of the newly generated embryonic 149,254,447 Illumina sequence reads, in addition to the adult male Illumina reads^46^, Trinity *in silico* read normalization^45^ was applied for removing redundant reads. The normalized Illumina data was assembled *de novo* into 820,838 contigs with a total number of 327,796,547 bp and a sequence length range from 201 bp to 17,100 bp (Supplementary Table S3).

Before the two pre-assemblies were combined into a hybrid assembly, potential contamination and redundancy were removed (Supplementary Table S3). These improved contigs of both pre-assemblies were clustered into combined into hybrid contigs using CAP3. To improve the overall quality of the hybrid assembly and to remove potential assembly artefacts, open reading frames with at least 5 bp mean coverage per base were only selected for the final reference assembly (Supplementary Table S3). This final reference assembly comprised 105,664 transcript isoforms and 92,284 unique transcripts, with an N50 of 1,571. The completeness analysis revealed 235 complete CEG’s (94.76%) and 10 partial CEG’s, resulting in an estimated gene completeness of 98.79% (245/248) and a BUSCO completeness of 82.02% (1,128 complete single-copy BUSCOs, 549 complete duplicated BUSCOs, 517 fragmented BUSCOs, 481 missing BUSCOs), thereby indicating a very complete representation of expressed genes which could be used as a reference.

### Annotation

The hybrid assembly was further annotated using the Trinotate Pipeline (v3.0.0) (https://trinotate.github.io/), including 1) capturing Blast homologies (BLASTX and BLASTP) against Uniprot-uniref90 database and Swissprot database (https://data.broadinstitute.org/Trinity/Trinotate_v2.0_RESOURCES/, both accessed March 2016), 2) protein domain identification using PfamA database (https://data.broadinstitute.org/Trinity/Trinotate_v2.0_RESOURCES/, accessed March 2016), 3) prediction of signal peptides using SignalP (v4), 4) prediction of transmembrane regions using tmHMM (v2), and 5) identification of rRNA transcripts using RNAMMER. Trinotate further retrieves various Kegg, GO, and Eggnog annotations from the Swissprot database.

A total of 122,769 annotations were retrieved for the hybrid assembly, of which 49,352 unique gene IDs have retrieved at least one annotation (Supplementary Table S4). We further sorted the annotations according to BLASTX homologies against the Uniprot-uniref90 database and analysed which species were most highly represented. Here, out of these 26,586 unique gene IDs with a Uniprot-uniref90 annotation based on BLASTX, *Zootermopsis nevadensis* proteins dominated these BLASTX results (7,182 contigs), which also reflects the phylogenetic distance to the other proteomes^37^ (Supplementary Fig. S1).

Gene Ontology (GO) analysis was further performed using the GOseq package adjusting for transcript length bias in deep sequencing data^68^ and using the GO annotation retrieved from the Trinotate annotation pipeline. GO terms were further summarized to generic GOSlim categories using the R package GOstats^69^.

### Read Abundance and Stage Specific Expression and Similarity

Transcript quantification revealed 105,665 (isoforms)/92,285 (genes) reference transcriptional active sequences, of which 6,794 unique genes were expressed throughout all embryonic stages (Supplementary Table S5). The major represented GO terms according to GOSlim of these “core” embryonic genes were genes associated with (1) anatomical structure development, (2) cellular nitrogen compound metabolic process, (3) biosynthetic process, (4) transport, and (5) small molecule metabolic process (Supplementary Fig. S2, Supplementary Table S5). For all downstream analyses, only read counts at the putative gene level were used. Distribution of expression patterns across the embryonic stages was further evaluated by dividing RPKM values into six bins and defining gene expression into low-(>0–5), moderate-(>5–50) and high-expression (>50). This revealed that in all embryonic stages the majority of transcripts are expressed at a low level (see Supplementary Fig. S3). In addition, starting from day 6 in the embryonic development more transcriptional active sequences could be detected.

**Figure 2:**
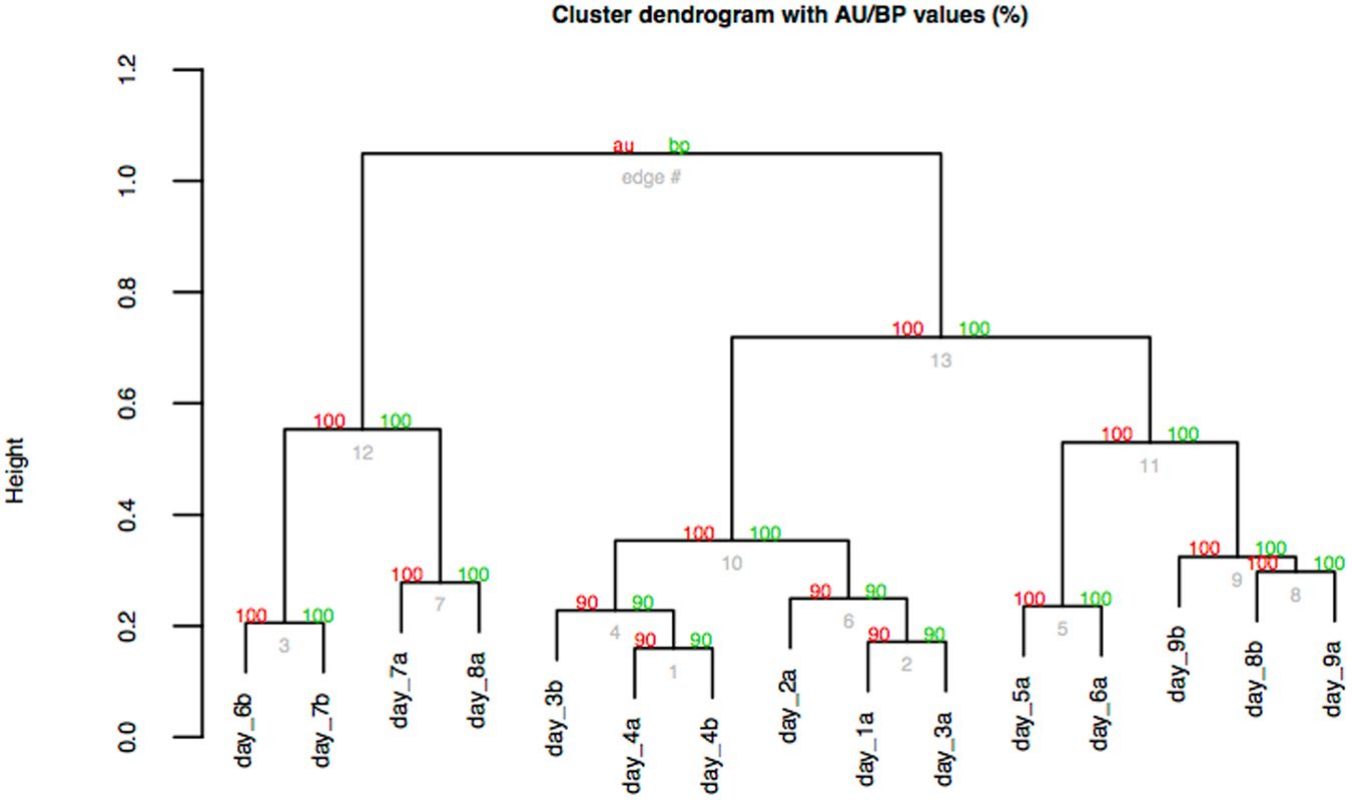
Hierarchical clustering of *I. elegans* embryonic expression patterns via multiscale bootstrap resampling. Analysis has been conducted on the filtered and normalized count matrix (27,027 unique genes). Bootstrap Probability (BP) and Approximately Unbiased (AU) values were computed for each of the clusters using complete linkage clustering based on Pearson correlation.

To measure the similarity of the samples covering all embryonic stages, the filtered and normalized count matrix (see Methods) was used for cluster bootstrapping analyses (10,000 iterations) using the R package PVClust (v.1.3-2)^70^ (Fig. 2). The bootstrap analysis provided statistical support for the sample relationships based on their gene expression and that the samples were differentiated according to embryonic stage status (*i.e*. day 1–4 versus day 5–6). The same differentiation between the samples and embryonic stage was revealed by multi-dimensional scaling (MDS) analyses (Supplementary Fig. S4). Here the samples were differentiated according to embryonic stage status (*i.e*. day 1–5/6 versus day 6/7–9) along dimension 1, while dimension 2 further separated the samples from day 6–8. In addition, both analyses revealed that individuals from the same developmental stages – as determined according to the days after oviposition – do not necessarily closely cluster together, indicating that there is variation amongst the individuals from the same embryonic stage. The ‘b’ sample of day 6 has a more similar gene expression to day 7 and 8 than to the ‘a’ sample of day 6, which clusters together with day 5. The same holds true for sample ‘b’ of day 3 which is more similar to day 4 while the other day three sample is more similar to day 1–2 (Fig. 2). The female damselfly’s oviposition behaviour could be an explanation for this variance between individuals of the same developmental stage according to oviposition. It was observed that the female in captivity lays the individual eggs during a long time span (>12 hours) that already accounts for a natural high variance in the development. This could be also further observed in the variable hatching times (9–10 days) of the embryos although kept under the same conditions which is known to strongly influence hatching times in general^71,72^. Furthermore, the comparable long time span and the relative low number of collected samples could not cover this existing natural high variance in the development between the individual embryos.

Consequently, our samples could not be treated as biological replicates and another complementary approach to compare expression between embryonic stages was adopted because differential expression studies require biological replicates for accuracy^73^. We therefore employed WGCNA, which is a topological-similarity based hierarchical clustering method that has been widely used in transcriptome studies^74–76^.

**Figure 3:**
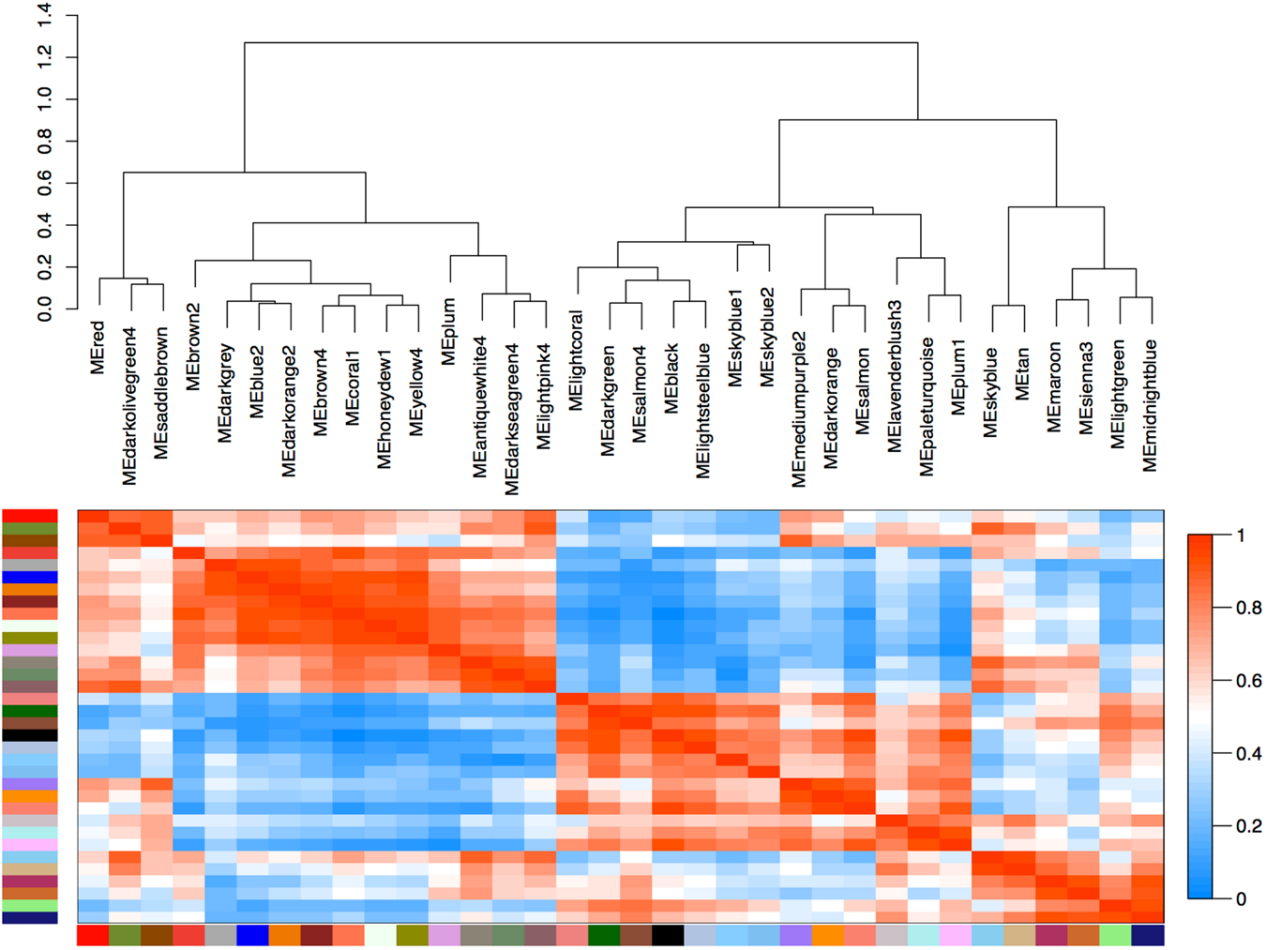
Module similarity by eigengene correlation of merged modules. Modules were merged based on a dissimilarity of module eigengenes (MEDissThres) of 0.1. This resulted into a total of 34 modules.

**Figure 4:**
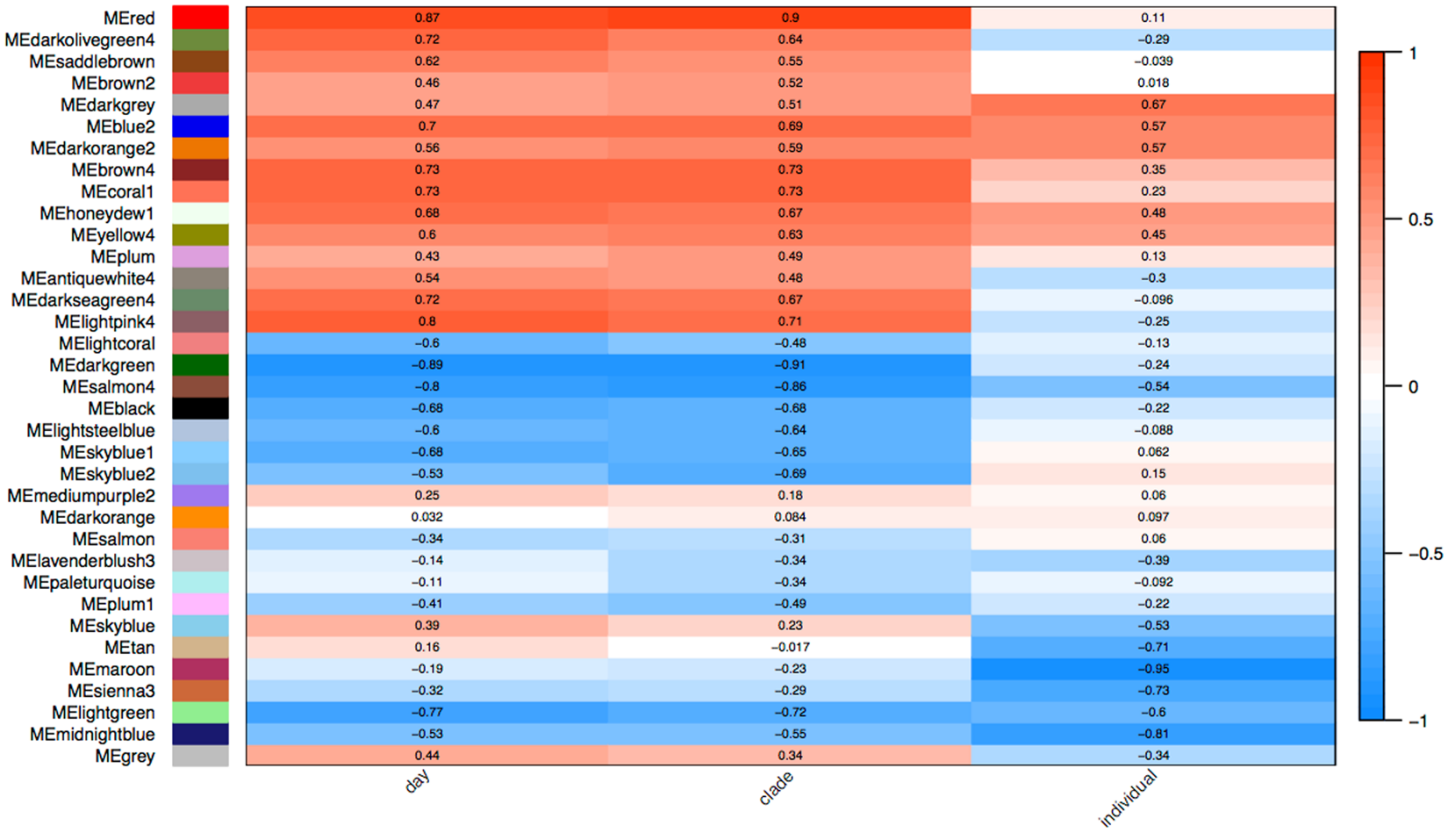
Module-‘trait’ association. Each row corresponds to a module. The correlation of the module with the corresponding ‘trait’ is provided in the associated cell. The FDR adjusted p-values as well as the gene number per module is provided in Supplementary Table S7.

A filtered count matrix comprising 27,027 genes was used for a step-by-step signed hybrid co-expression network approach. We also used BLASTX to locally compare the 27,027 filtered genes against all Arthropoda protein sequences (NCBI non-redundant protein (nr) database February 2016; E-value cutoff ≤10^−3^) (Supplementary Table S6). For the signed hybrid co-expression network the minimum module size was adjusted to 50 as we expected high biological variance between our samples as already indicated by the clustering and MDS analyses (Fig. 2 and Supplementary Fig. S4). However, subsequent quantification of module similarity revealed that DynamicTreeCut^66^ might have identified modules which are very similar (Supplementary Fig. S5). Therefore modules were merged based on module eigengene correlations of 0.9 (MEDissThres = 0.1). Although module similarity of the 34 merged modules based on eigengene correlation is for some modules still high, the dissimilarity of module eigengenes (MEDissThres) was set to a small value (0.1) because the samples are fairly biologically different and consequently we expected a large number of resulting modules (Fig. 3). The gene expression of the merged modules covering the embryonic development is shown in Supplementary Fig. S6. The 34 module eigengenes for the 34 merged modules were correlated with specific sample ‘traits’ (Fig. 4). These ‘traits’ were defined as 1) day: the embryonic stage as defined as the day after oviposition, 2) clade: clade definition according to the sample relationships based on the bootstrap and the MDS analysis, and 3) individual: ‘a’ or ‘b’ of the biological replicate (see Supplementary Table S7).

**Figure 5:**
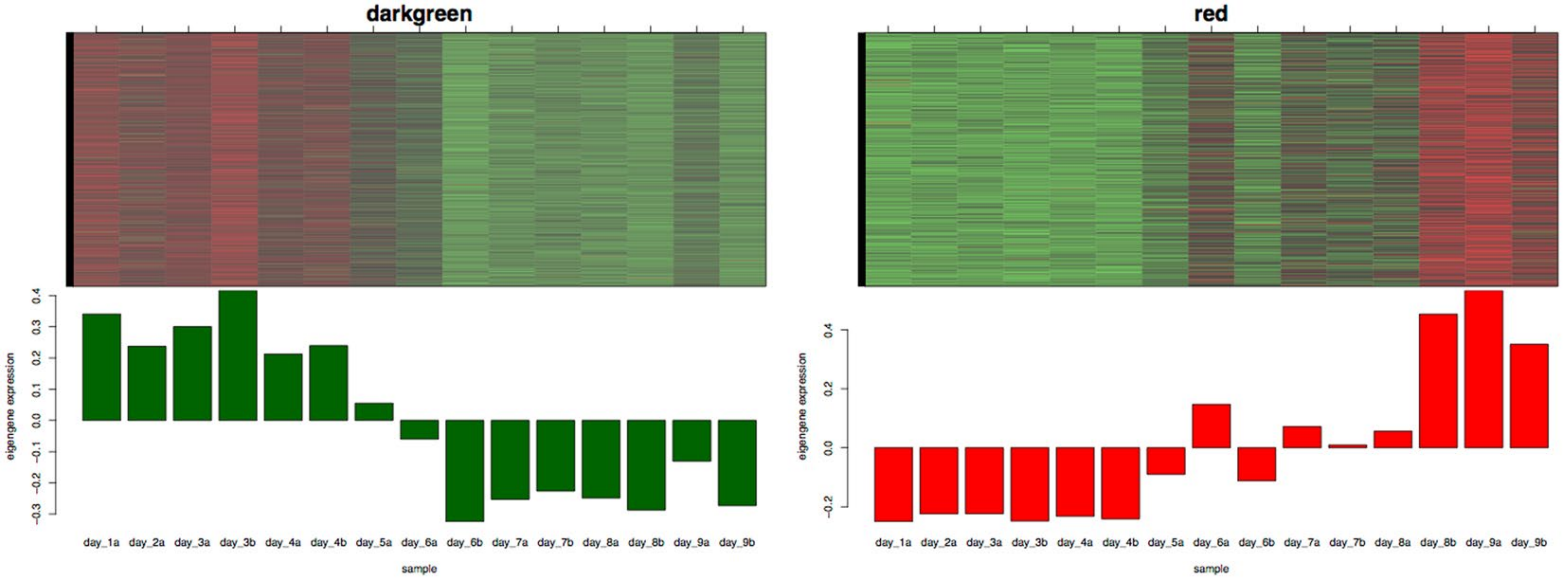
Heatmap and eigengene expression profiles of highest day significant modules. The y axis indicates the value of the module eigengene, the x axis the development and sample type. (**A**) The darkgreen module comprises 1,303 genes and a correlation coefficient of r = −0.89 with a FDR adjusted p-value 0.00032. (**B**) The red module comprises 635 genes and a correlation coefficient of r = 0.87 with a FDR adjusted p-value 0.00039.

Notably, 15 out of the 34 co-expression modules were significant correlated for day and clade (FDR adjusted p-values < 0.05 (Supplementary Table S8)). Day was most strongly correlated with the darkgreen and the red module, although with opposite directions (r = −0.89, r = 0.87 and both with FDR adjusted p-value < 4 × 10^−4^, respectively) (Fig. 5). For these two modules the 30 most highly expressed genes were identified because they might provide insights into important processes during these developmental stages. Notably, in the darkgreen module, with a high eigengene expression during developmental stages day 1–day 4, the most abundant transcripts were ribosomal proteins, further reflecting the fact that ribosome formation is a significant activity during the earliest stages of insect embryogenesis^77^ (Supplementary Table S9). Additionally highly expressed genes were related to DNA replication (Mcm7), transcription regulation (Hrp65), and mRNA processing (Protein DEK). We further identified Geminin among the 30 most highly expressed in the darkgreen module, which plays a role in DNA replication, in anaphase and in neural differentiation^78^. In contrast, in the red module, with a high eigengene expression during developmental stages day 8–day 9, most of the highly expressed transcripts were muscle function related proteins such as Muscle LIM protein (Mlp), Troponin (Tpn) and Tropomyosin (Tm) and Actin (Act). Also detected were proteins involved in the formation of cuticle (cuticle protein 21-like, endochitinase).

A recent study has revealed significant sex-biased gene expression in *I. elegans* adults^79^. And although it has been shown that the amount of sex-biased gene expression tends to increase during development, with low levels in embryonic stages and high levels in sexually mature adults^80,81^, we explored if the identified modules could be a result of sex-biased gene expression. Under the assumption that the embryos would developed in m males and n females (with m, n, >0 and m + n = 15), we evaluated if specific modules were related to the ‘trait’ sex by considering all 16,383 possible partitions of the 15 sample in two groups (1–2, male-female, female-male respectively) and testing their correlation with the identified gene expression modules. We found only the skyblue2 module to be significantly correlated at the 0.01 confidence level after Bonferroni correction (p-value = 2.82 × 10^−8^) to one of these sex combinations after Bonferroni-correction on a 0.01 nominal p-value correction (p-value = 2.92 × 10^−11^) (Supplementary Fig. S7). The skyblue2 module comprises 107 genes and the annotation against Arthropoda protein sequences (NCBI non-redundant protein (nr) database, assessed February 2016) (Supplementary Table S10) and the GO analysis revealed overrepresented genes involved into the structural constituent of cuticle (GO:0042302) and serine-type exopeptidase activity (GO:0070008). Interestingly, previous studies have shown sex-dependent differential expression of proteins involved in the structural constituent of cuticle, e.g. cuticle composition^82,83^. Nevertheless, based on these analyses we concluded that the identified clusters significant correlated for day and clade within *I. elegans* embryogenesis were not a result of sex-biased gene expression.

We further used the WGCNA measure of intramodular connectivity (kME) to identify intromodular hub genes in all 15 significantly day- and clade-related modules. Expression profiles of hub genes represent that of the entire module^84^ and has been found to have more biologically relevant information than whole-network hub genes when considering gene co-expression networks^85^. In total, 3,452 hub genes in 15 modules were identified (kME > 0.9, p-value < 10^−6^) (Supplementary Table S11).

**Figure 6:**
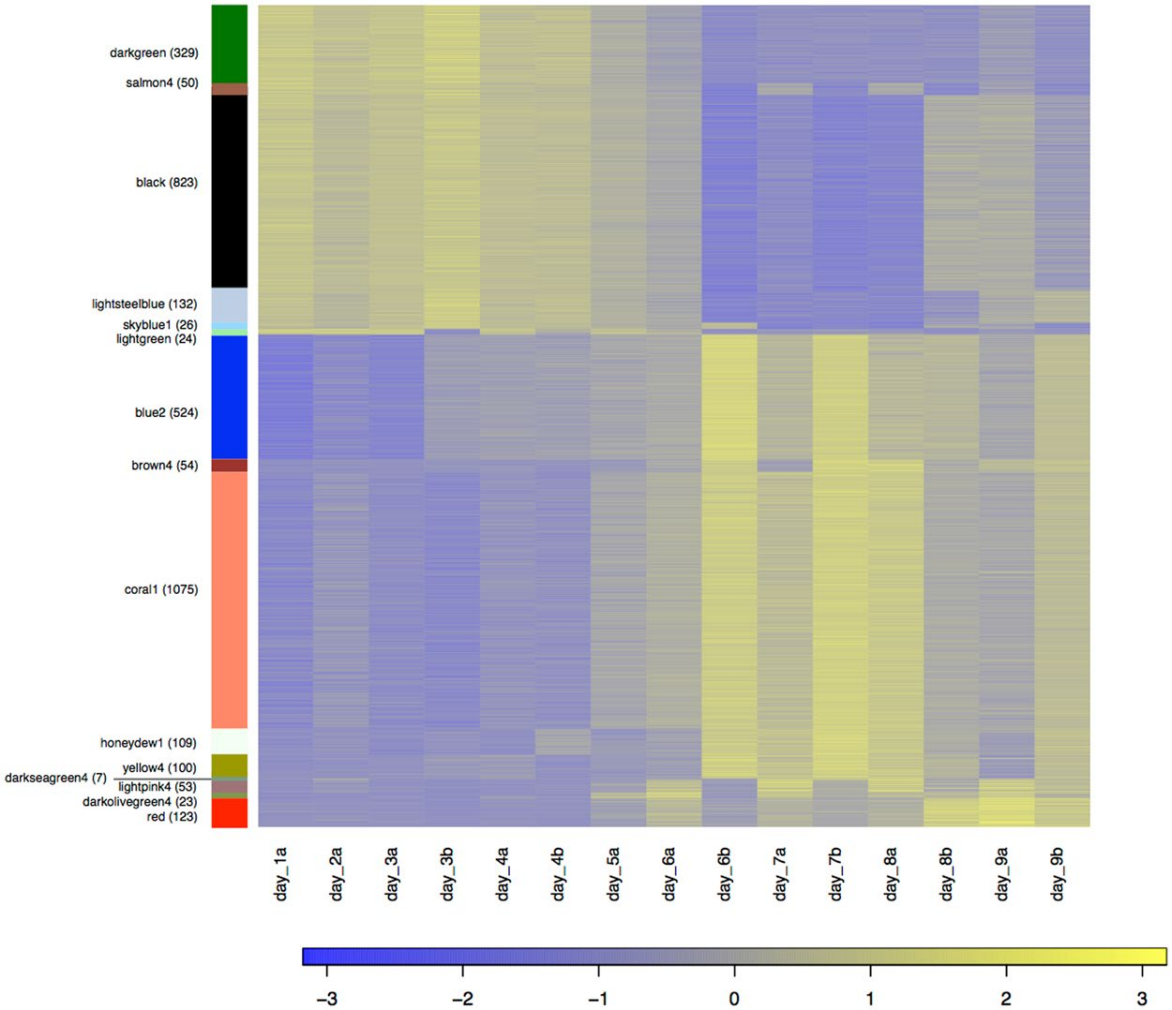
Heatmap of the 3,452 identified hub genes in the significant day and clade module. Each row of the heatmap represents a single gene and genes are grouped according to their module membership. The number of hub genes in each module is given in parenthesis. Modules are sorted according to their module similarity (see also Fig. S5). Expression patterns of all hub genes are visualized as Z-scores using the log2 TMM-normalized FPKM values.

**Figure 7:**
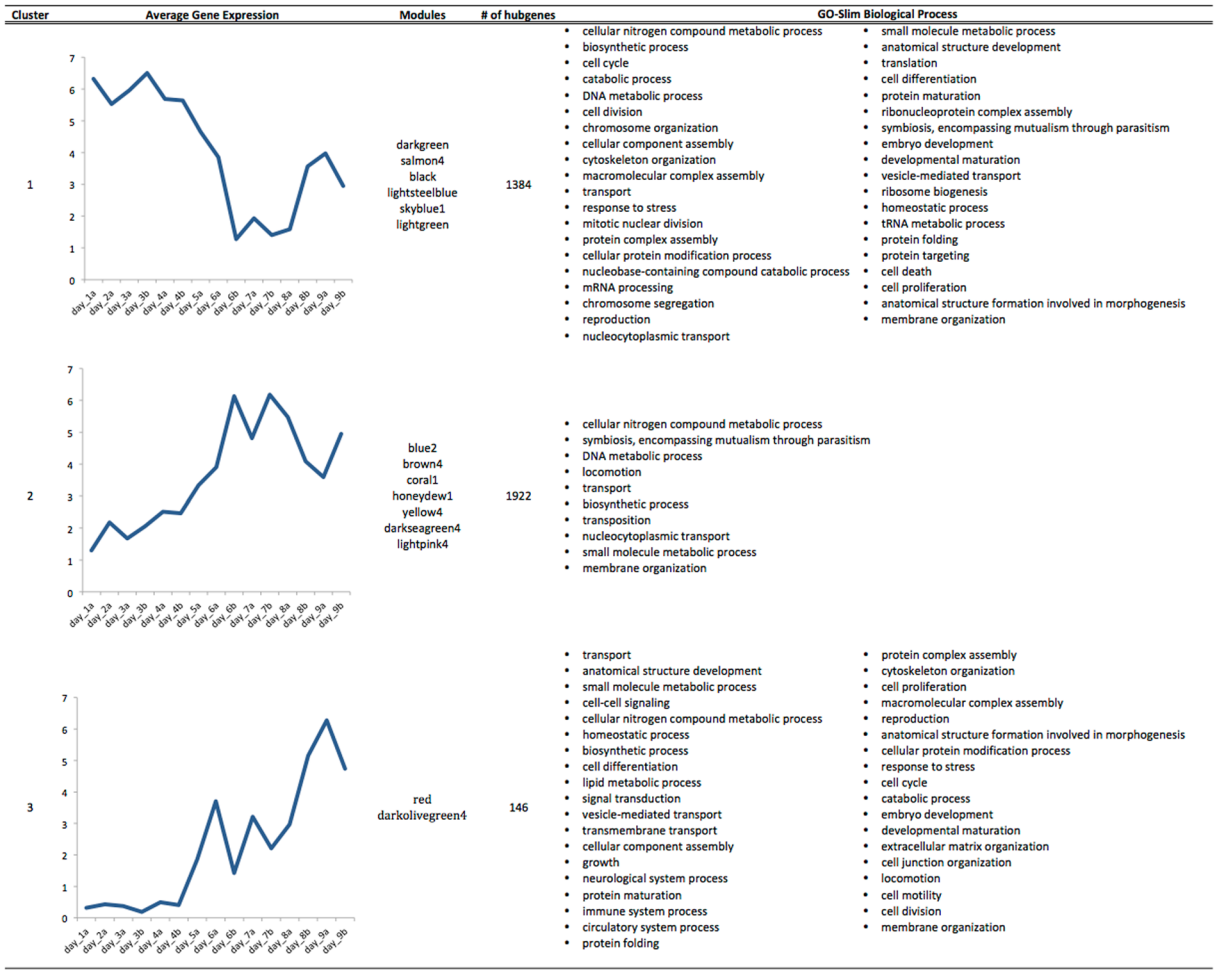
GOSlim categories for over-represented biological processes in each of the three clusters. The average gene expression (TMM log2) is based on the identified hub genes in the corresponding modules.

A heatmap of the identified hub genes is shown in Fig. 6. Based on this, three clusters of similar gene expression could be observed (see also Fig. 7):

- Cluster 1: modules darkgreen, salmon4, black, lightsteelblue, skyblue1, lightgreen; gene expression up-regulated early during embryogenesis (day 1-day 4/5), followed by a down-regulation after mid-embryogenesis (day 6-day 7) and an up-regulation again during late embryogenesis (day 8-day 9).
- Cluster 2: modules blue2, brown4, coral1, honeydew1, yellow4, darkseagreen4, lightpink4; gene expression antagonistic to cluster 1. Gene expression down-regulated early in embryogenesis (day 1-day 4/5), followed by an up-regulation after mid-embryogenesis (day 6–day 7) and a down-regulation again during late embryogenesis (day 8–day 9).
- Cluster 3: modules red and darkolivegreen4; gene expression up-regulated from day 5 on and the highest genes expression during late embryonic stages (day 8–day 9).

For the identified hub genes, statistically over-represented GO terms in a given gene list were identified using the Benjamini-Hochberg correction (p-value < 0.05) relative to the reference set of the 27,027 genes. These statistically over-represented GO terms were further summarized to generic GOSlim categories (Fig. 7 and Supplementary Table S12).

We further analysed the expression dynamics of conserved signalling pathways as well as key developmental genes. A list of *D. melanogaster* genes from Flybase according to the signalling pathways and developmental processes as assigned by QuickGO (http://www.ebi.ac.uk/QuickGO/, assessed February 2016) was used as a query to identify homologous sequences in the *I. elegans* transcriptome. The developmental pathways included embryonic axis formation (GO:0000578), regulation of JAK-STAT cascade (GO:0046425), TGFbeta receptor signalling pathway (GO:0007179), Notch signalling pathway (GO:0007219), hedgehog signalling pathway (GO:0007224), sex determination (GO:0007530), Wnt signalling pathway (GO:0016055), and segmentation (GO:0035282). Transcripts with a blast hit to *Drosophila* (E-value cutoff ≤ 10^−3^) were then used in a reciprocal blast analysis using BLASTX against all Arthropoda protein sequences (NCBI non-redundant protein (nr) database, assessed February 2016) to establish orthology. Blast results were manually selected for ortholog matches and tabulated (Supplementary Table S13). The expression dynamics across the embryonic development of selected developmental genes are further shown in Supplementary Figs S8–S15.

### Gene Expression Divergence In Relation to Embryonic Developmental Stages

The embryogenesis of hemimetabolous insects can be broadly divided into germ band formation, anatrepsis, intertrepsis (or germband stage) and katatrepsis^18,86,87^. During the earliest embryonic stages, proliferation of the germ band is followed by penetration into the yolk mass and differentiation of protocephalon (wide anterior portion of the embryo) and protocorm (narrow posterior region) occur. In long germ types, which are only found in multiple clades within the Holometabola, all segments develop simultaneously at the blastoderm stage^88^. Contrary, Odonata display an intermediate germ type^89^ where an anterior stretch of the germ anlage subdivides rapidly to yield the anterior segments (protocephalon), whereas the remaining segments are added successively^90^. With abdomen elongation and segmentation, anatrepsis – invagination of the embryo into the yolk and posterior movement of the head – starts^86^. Following anatrepsis, the abdomen further elongates during intertrepsis and has to curl back towards the head of the embryo. During this stage the appendage formation starts and thoracic segments are more clearly defined^18^. Intertrepsis is followed by katatrepsis, a 180° rotation of the whole insect embryo within the egg by reorganization of the extraembryonic membranes that repositions the embryo^86^. The entire process of movement during embryonic development within the egg is also summarized as blastokinesis in concert with morphogenetic movements of the two extraembryonic membranes and occurs only in hemimetabolous insects: for review see also Panfilio^86^. In all Odonata, katatrepsis takes place midway in embryonic development and lasts only a few hours^17^. In previous detailed histological observation the same was also observed in *I. elegans* if kept under different temperature conditions^91^ (Simon *et al*., unpublished data). Ando^17^ also described the stages before katatrepsis (revolution) as pre-revolutionary stages and after katatrepsis as post-revolutionary stages.

The pre-revolutionary stages are mainly covered by cluster 1 which show highest expression levels from day 1 to day 4/5, followed by a later moderate up-regulation of the genes during late maturation of the embryo before hatching of the nymphs (day 8–day 9). This cluster was dominated by signatures of cell cycle (GO:0007049), cell division (GO:0051301), cell differentiation (GO:0030154) and mitotic nuclear division (GO:0007067) (Fig. 7 and Supplementary Table S12). This likely reflects an extensive reproduction of embryonic cell mass, pattern formation and regional specification that occurs during early embryonic stages until katatrepsis. This was further reflected in the expression dynamics of conserved signalling pathways as well as key developmental genes. For example, genes involved in axis formation and segmentation showed a clear down-regulation around mid-embryogenesis (Supplementary Figs S8 and S12). Here, for example Delta, which has a role in the proper morphogenesis of body segments and posterior elongation^15^ and hunchback, which plays a role in segment patterning^92^, could be identified (Supplementary Fig. S8). Several homeobox genes, e.g. homothorax, proboscipedia, ultrabithorax, LIM/homeobox protein Lhx9; and essential transcription factors, e.g. Transcription factor SOX-2, Transcription factor Sox-6, POU domain class 6 transcription factor 2, were found in the final transcripts (Supplementary Table S13), however they were not included in the identified hub genes due to their low expression levels. Cluster 1 was also enriched for genes involved in mRNA processing (GO:0006397), translation (GO:0006412), ribonucleoprotein complex assembly (GO:0022618) and ribosome biogenesis (GO:0042254) and highlight the rapid succession of cell cycles associated with chromatin replication and initiation of transcription and translation for embryo patterning^93^.

Cluster 3 harbours transcriptional active genes during post-revolutionary stages with sequential up-regulation from day 5 on and the highest gene expression during late embryonic stages (day 8/9). This cluster includes markers for neurological system process (GO:0050877), immune system process (GO:0002376), and circulatory system process (GO:0003013). Combination of specific activity of cell cycle markers different from the ones identified in cluster 1 indicates the final differentiation processes for maturation of the embryo prior to hatching. In addition, transcriptional active genes involved in cell-cell signaling (GO:0007267), homeostatic process (GO:0042592), and signal transduction (GO:0007165) reflect the peak time of organogenesis, in accordance with the observation of formation of the compound eye, differentiation of the tracheal system, and completion of heart development and muscle formation^17^. For example we identified Slit, an important regulator of axon guidance^94^, and Cubilin for functional maturation of nephrocytes and intestines^95^. High expression of genes involved in muscle structure and function such as Muscle LIM protein, Troponin, Tropomyosin, Myosin and Actin further indicates the maturation of the muscular system for active movement shortly before hatching. This is in agreement with the observation of Ando^17^ that the formation of musculature first takes place shortly before katatrepsis and makes rapid progress after the dorsal closure.

Interestingly, a major shift in gene expression was detected around mid-embryogenesis, presumably after katatrepsis (~day 6) and during the early post-revolutionary stages. This was reflected by cluster 2, which contrary to cluster1, showed elevated expression levels of marker genes from day 6 on. Peak expression appeared at days 6–8 and these stages were also clearly separated from the other developmental stages based on the cluster bootstrap analysis (Fig. 2) and the MDS analyses (Supplementary Fig. S4). The up-regulated genes during these stages comprised markers for locomotion (GO:0040011), transport (GO:0006810) and membrane organization (GO:0061024) and harbours mainly hypothetical and uncharacterised proteins (Supplementary Table S6). In addition, this cluster comprised several transposable elements (TEs), like DNA transposon Mariner and piggyback, and several newly expressed reverse transcriptases. TEs are known to occupy different portions of insect genomes and account for the huge variety in insect genome sizes^96^. In recent years there is increasing evidence that TEs play vital roles in regulation of gene expression by remodeling the chromatin conformation, by inserting into promoters or enhancers and providing binding sites for transcription factors^97–99^. In addition, TEs are known to play a role in insect embryonic development^100^, in phenotypic plasticity^101^, and diapause^102^. Indeed, active transcription of TEs has been detected at various stages of development and have a major role in generating intraspecies variation^103^. Recently, high transcriptional activity of TEs in the egg stage of the migratory locust, *Locusta migratoria*, was detected^101^. We detected the increase of TE expression around day 6 after down regulation of the DNA (cytosine-5)-methyltransferase 1 (DNMT1). DNMT1 was detected in module salmon4, which is part of cluster1 (Supplementary Table S6). Down-regulation of TEs occurred when DNA (cytosine-5)-methyltransferase 1 was again up-regulated after day 7/8. This observation is in agreement with previous studies where TE suppression is directly linked to increased DNA methylation activity^103^, although there is also recently increasing evidence for self-regulation of TEs^104^. The functional role of TE activity in mid-embryonic stages can only be speculated on and so far no comparable data exists to further verify an up-regulation of transposable elements after mid-embryogenesis (katatrepsis). One possible explanation for TE usage during embryonic development could be the inactivation of genomic regions, important for early embryonic regulation, by insertions and deletions of TEs as an alternative silencing mechanism, other than DNA methylation. This would be in agreement with our observation that cluster 1 and cluster 2 are exactly contrary transcriptionally active. So far, these data remain preliminary and the clarification of the overall role of TEs in insect embryonic development demands more detailed research.

In summary, the identified temporal cluster activities mirror the timeline for developmental progression. In contrast to Holometabola, *I. elegans* embryos develop directly into the final patterned pterygote Bauplan. Early axial/spatial progenitor establishment is mediated through cluster 1 transcript activity and after the appendages are established in adaptation to the aquatic- and terrestrial life cycle, the embryo undergoes katatrepsis/revolution. Differentiation of the muscular system, organs and outgrowth of appendages is later on governed by overlapping activities of cluster 2 and cluster 3 from mid embryonic stages on. While cluster 3 activity peaks during late embryonic stages when the embryo undergoes maturation, we detect a second onset of cluster 1 activity, shortly before hatching of the individuals. This indicates that final differentiation processes of *I. elegans* depend on early embryonic genes for very late embryonic developmental specification.

## Conclusion

In this study, we present the first comprehensive embryonic transcriptome of a hemimetabolous insect, the damselfly *I. elegans*. Using a single-embryo sequencing approach we were able to elucidate the transcriptional divergence of pre- and post-revolutionary embryonic stages highlighting the transcriptional complexity during insect embryogenesis. During pre-revolutionary stages, reflecting early embryogenesis until katatrepsis, transcriptional active genes were characterised for their biological functions in cell cycle, mitosis and differentiation. In addition, genes involved in signalling pathways and key development processes were enriched during these early embryonic stages. This is indicative for cell mass production for germ-band elongation and subsequent early pattern formation. During post-revolutionary stages, we identified up-regulated genes related to late embryonic development such as active movement and signal transduction for sensory perception. For the transit to the nymphal stage, we further observed activation of genes involved in circulatory, immune and neurological system maturation. The increased activity of transposable elements of different classes during mid-embryogenesis could indicate a previously unknown mechanism for developmental gene regulation. Evidently, more comprehensive embryonic transcriptomic studies of hemimetabolous insect are needed for elucidating a potential role of transposable elements and their correlation to post-revolutionary embryogenesis.

## Electronic supplementary material

Supplementary Figures S1-S15

Table S1

Table S2

Table S3

Table S4

Table S5

Table S6

Table S7

Table S8

Table S9

Table S10

Table S11

Table S12

Table S13
