# Supplementary material for: Comparative transcriptomics reveal developmental turning points during embryogenesis of a hemimetabolous insect, the damselfly Ischnura elegans
Source: Sci Rep. 2017 Oct 19;7:13547. doi: 10.1038/s41598-017-13176-8 (PMC5648782; doi:10.1038/s41598-017-13176-8)
Supplement: Supplementary file 1 — Supplementary Figures S1-S15 [file 41598_2017_13176_MOESM1_ESM.pdf]

Comparative transcriptomics reveal developmental turning points during embryogenesis of a hemimetabolous insect, the damselfly *Ischnura elegans*

Sabrina Simon[1,2], Sven Sagasser[3], Edoardo Saccenti[4], Mercer R. Brugler[2,5], M. Eric Schranz[1], Heike Hadrys[2,6,7], George Amato[2], Rob DeSalle[2]

1 – Biosystematics Group, Wageningen University & Research, Droevendaalsesteeg 1, 6708 PB Wageningen, The Netherlands
2 – Sackler Institute for Comparative Genomics, American Museum of Natural History, Central Park West and 79th St., New York, NY 10024, USA
3 – Ludwig Institute for Cancer Research, Karolinska Institutet, 17177 Stockholm, Sweden
4 – Laboratory of Systems and Synthetic Biology, Wageningen University & Research, Stippeng 4, 6708 WE Wageningen, The Netherlands
5 – Biological Sciences Department, NYC College of Technology, City University of New York, 300 Jay Street, Brooklyn, New York 11201, USA
6 – ITZ, Ecology&Evolution, University of Veterinary Medicine Hanover, Buenteweg 17d, D-30559 Hannover, Germany
7 – Yale University, Department of Ecology & Evolutionary Biology, 165 Prospect Street, New Haven, CT 06511, USA

Correspondence:
Sabrina Simon, Biosystematics Group, Wageningen University & Research, Droevendaalsesteeg 1, 6708 PB Wageningen, The Netherlands. E-Mail: sabrina.simon@wur.nl

Supplementary Tables

All Supplementary Tables are provided as separate Supplementary Dataset File.

Table S1: Developmental stage and number of embryos used for 454 and Illumina sequencing approach.

Table S2: Number of raw reads for each library and resulting reads after trimming and cleaning.

Table S3: Statistics of pre-assemblies and final hybrid assembly.

Table S4: Trinotate annotation of the hybrid assembly. The final reference transcriptome is available in the TSA database under BioProject PRJNA401426.

Table S5: Gene expressed in all embryonic stages. Provided are the RPKMs and the annotation against the arthropod database.

Table S6: Blast result of the 27,027 filtered genes (used for WGCNA) against the Arthropoda database.

Table S7: 'Trait' definition for the WGCNA analyses.

Table S8: FDR adjusted p-values for modules and correlation to the 'traits'. Significant modules are highlighted in red.

Table S9: Annotation of the 30 most highly expressed genes for the two most significant modules (darkgreen and red) correlated with the 'trait' day.

Table S10: Blast results of transcripts of the skyblue2 module against the Arthropoda database.

Table S11: Eigengene-based connectivity and student asymptotic p-value for the 3,452 hub genes in 15 significant modules including annotation against the Arthropoda database.

Table S12: GOslim categories for genes identified in the three clusters.

Table S13: Blast results of transcripts against *Drosophila* developmental pathway genes. Only blast hits with a reciprocal blast hit using BLASTX against all Arthropoda protein sequences to establish orthology are provided.

Supplementary Figures

Figure S1: Species distribution of the best BLASTX hit against the Uniprot-uniref90 database. ZOONE: *Zootermopsis nevadis* (dampwood termite), ACYPI: *Acyrthosiphon pisum* (pea aphid), TRICA: *Tribolium castaneum* (flour beetle), PEDHC: *Pediculus humanus* (body louse). The majority (7,182 contigs) share the highest similarity with the proteome of *Zootermopsis nevadensis*.

Figure S2: GOslim categories for genes expressed in all embryonic stages. The y axis indicates the number of GO terms assigned to corresponding GOSlim. Shown are only the top 20 GOSlim categories, all categories and frequencies are listed in TableS5.

Figure S3: Distribution of expression categories across *I .elegans* embryonic stages. The y axis shows the number of expressed contigs and the x axis represents RPKM values.

Figure S4: Multi-dimensional scale plot of all *I. elegans* embryonic samples. Analysis has been conducted on the filtered and normalized count matrix (27,027 unique genes).

Figure S5: Module similarity by eigengene correlation. Analysis has been conducted on the filtered and normalized count matrix (27,027 unique genes) using WGCNA. The relationships of the initial 78 identified modules have been analysed using the eigengenes as representative profiles and quantify module similarity by eigengene correlation. Note the clustering of the module eigengenes and their high similarity (above). Also the heatmap shows (below) shows the similarity of several modules based on the eigengene correlation.

Figure S6: Gene expression across the embryonic development of *I. elegans* within the merged modules.

Figure S7: Heatmap and eigengene expression profiles of potential sex significant module. The y axis indicates the value of the module eigengene, the x axis the development and sample type. The skyblue2 module comprises 107 genes and a correlation coefficient of r = 0.98 with a Bonferroni adjusted nominal p-value 2.92×10^{-11}. Partition: 1-1-2-2-2-2-1-1-1-1-1-1-1-1-1 (1-2, male-female, female-male respectively)

Figure S8-S15: Expression dynamics of several developmental processes during *I. elegans* embryogenesis**.**

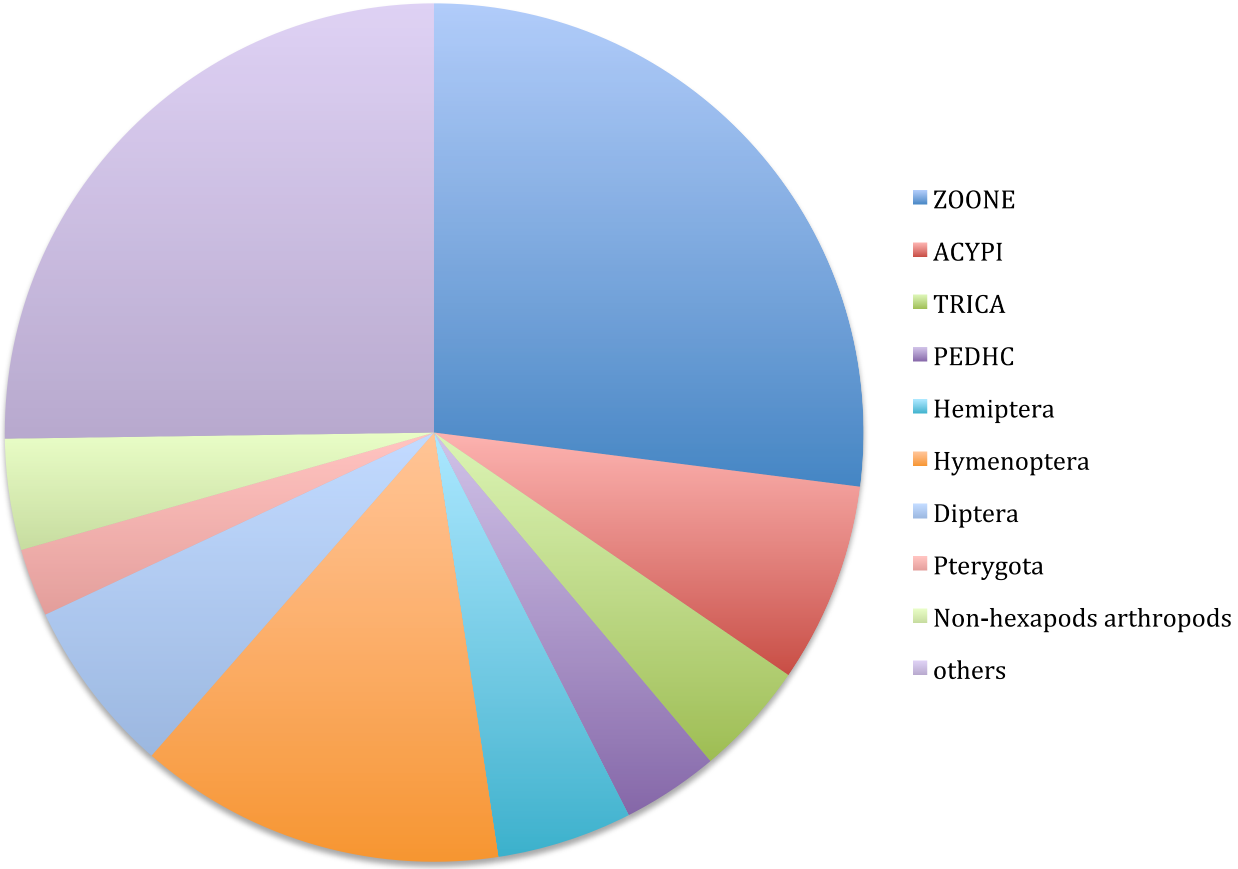

Figure S1: Species distribution of the best BLASTX hit against the Uniprot-uniref90 database. ZOONE: *Zootermopsis nevadis* (dampwood termite), ACYPI: *Acyrthosiphon pisum* (pea aphid), TRICA: *Tribolium castaneum* (flour beetle), PEDHC: *Pediculus humanus* (body louse). The majority (7,182 contigs) shares the highest similarity with the proteome of *Zootermopsis nevadensis*.

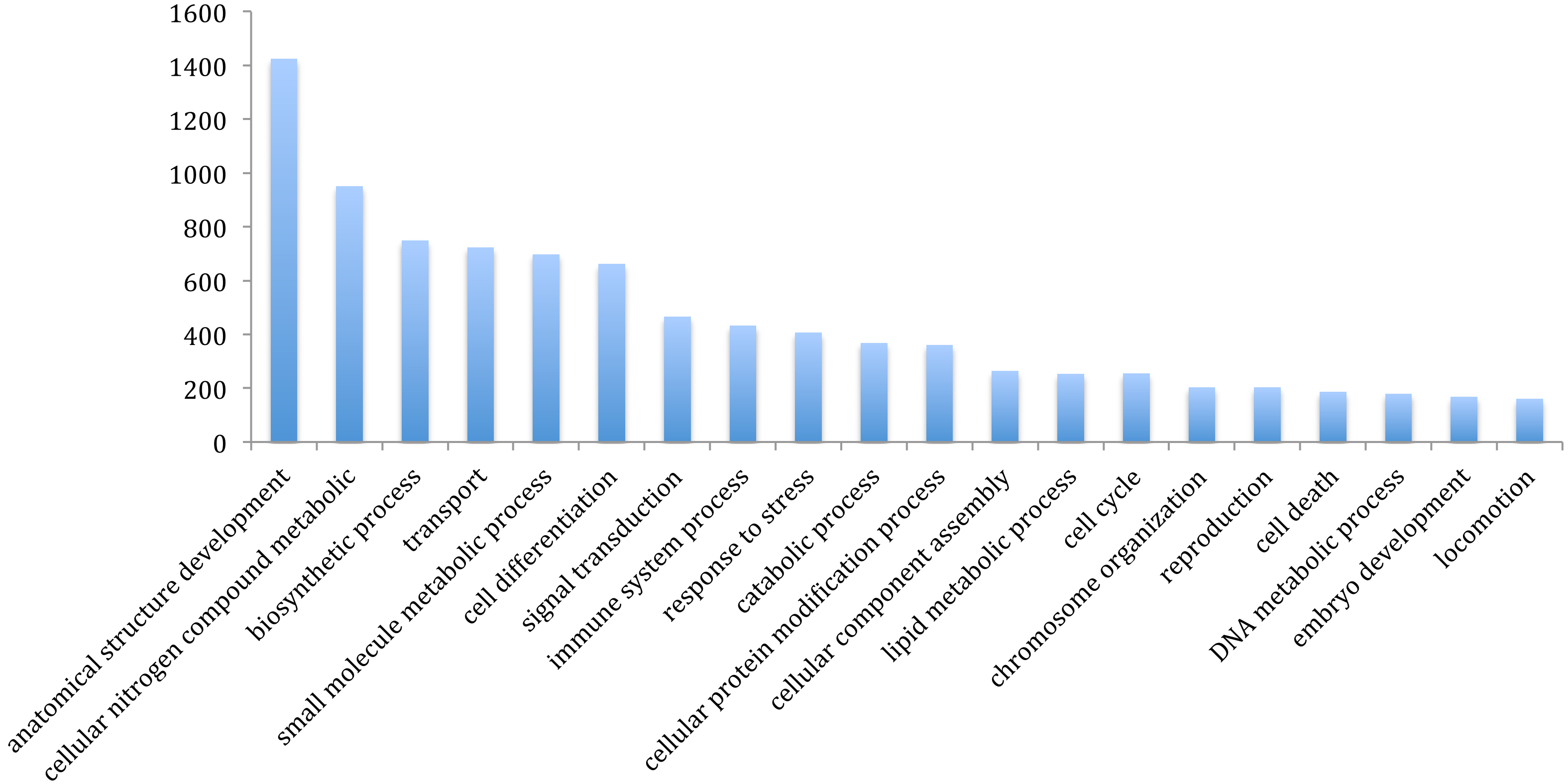

Figure S2: GOslim categories for genes expressed in all embryonic stages. The y axis indicates the number of GO terms assigned to corresponding GOSlim. Shown are only the top 20 GOSlim categories, all categories and frequencies are listed in Supplementary Table S5.

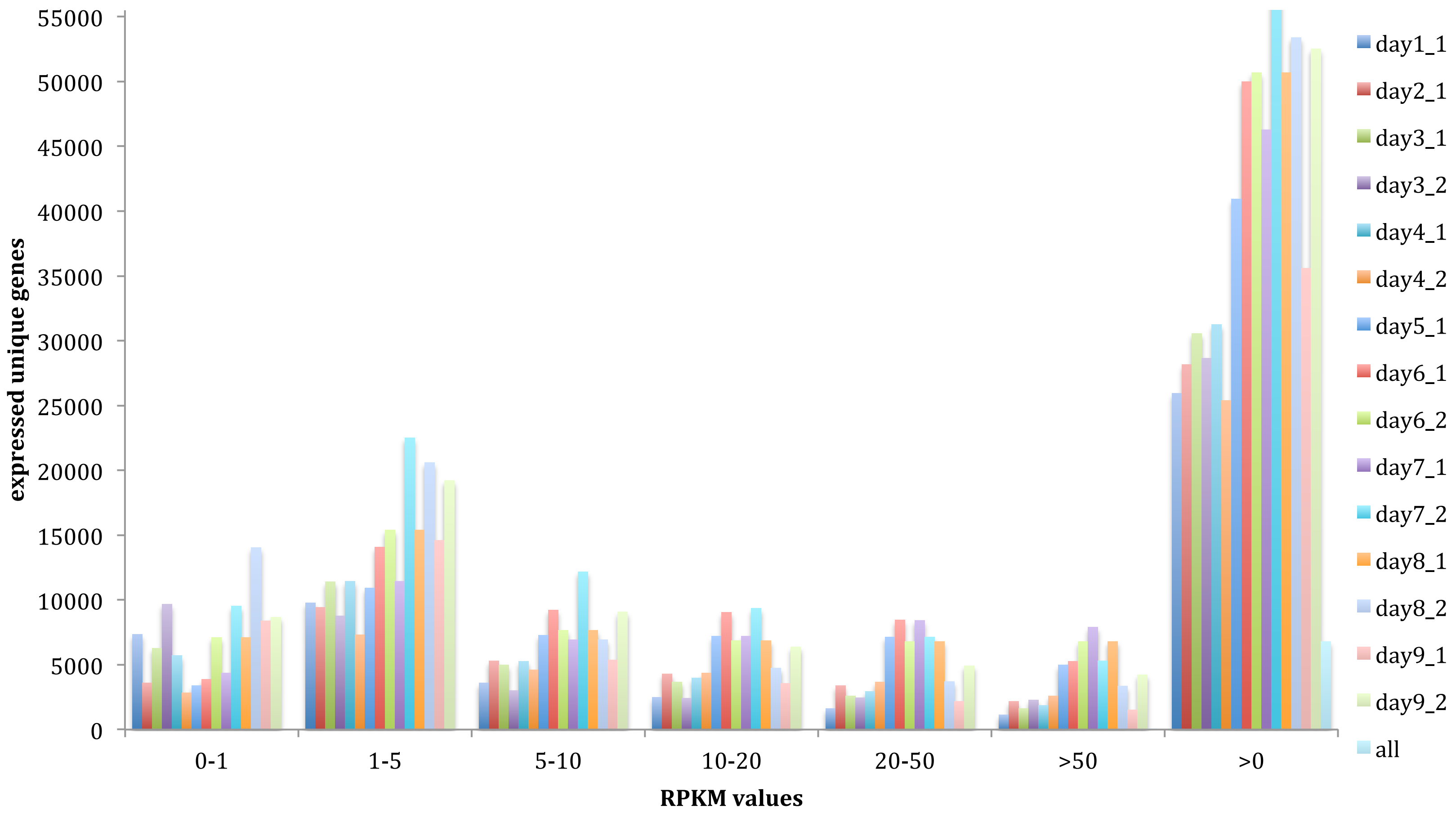

Figure S3: Distribution of expression categories across *I. elegans* embryonic stages. The y axis shows the number of expressed contigs and the x axis represents RPKM values.

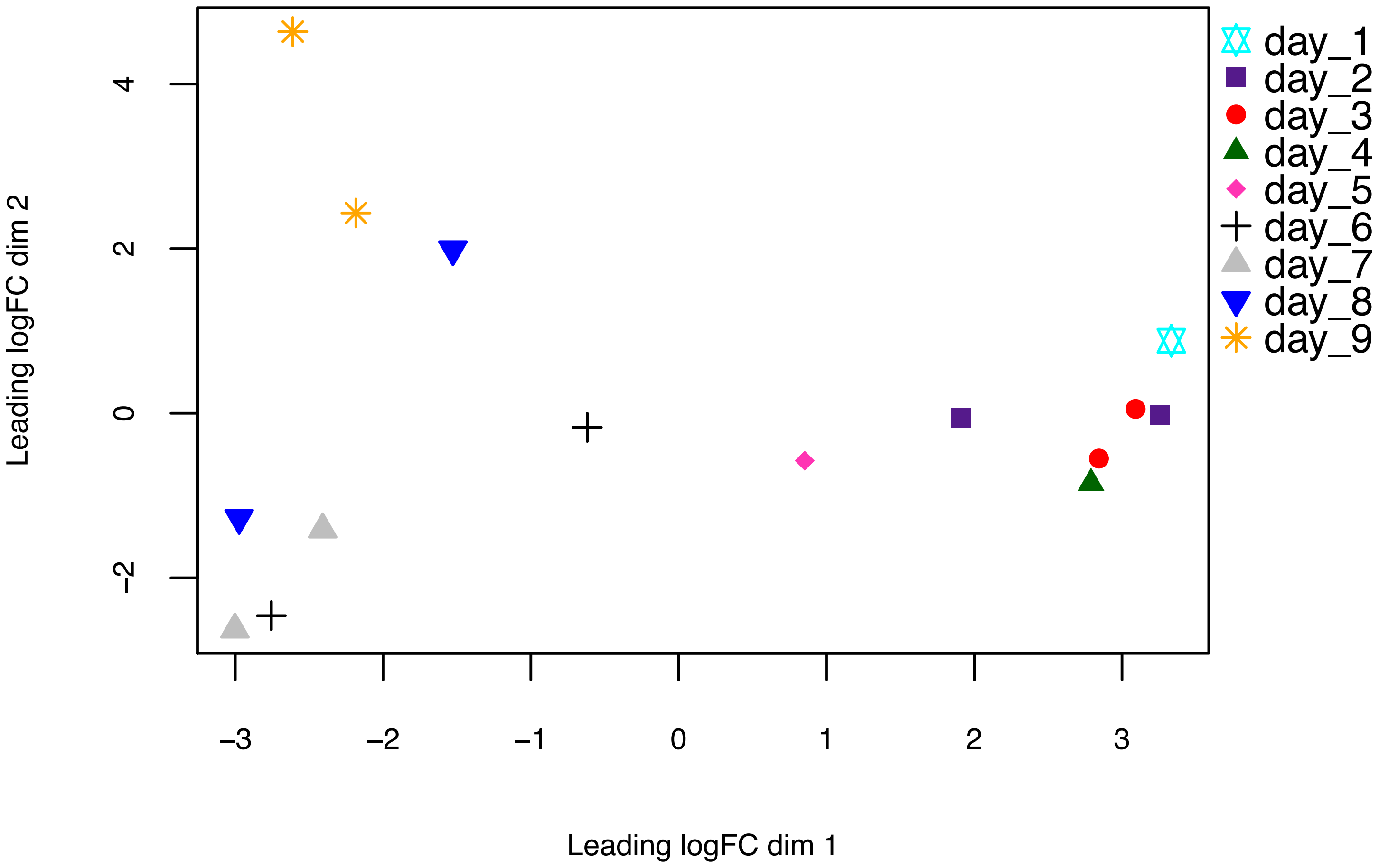

Figure S4: Multi-dimensional scale plot of all *I. elegans* embryonic samples. Analysis has been conducted on the filtered and normalized count matrix (27,027 unique genes).

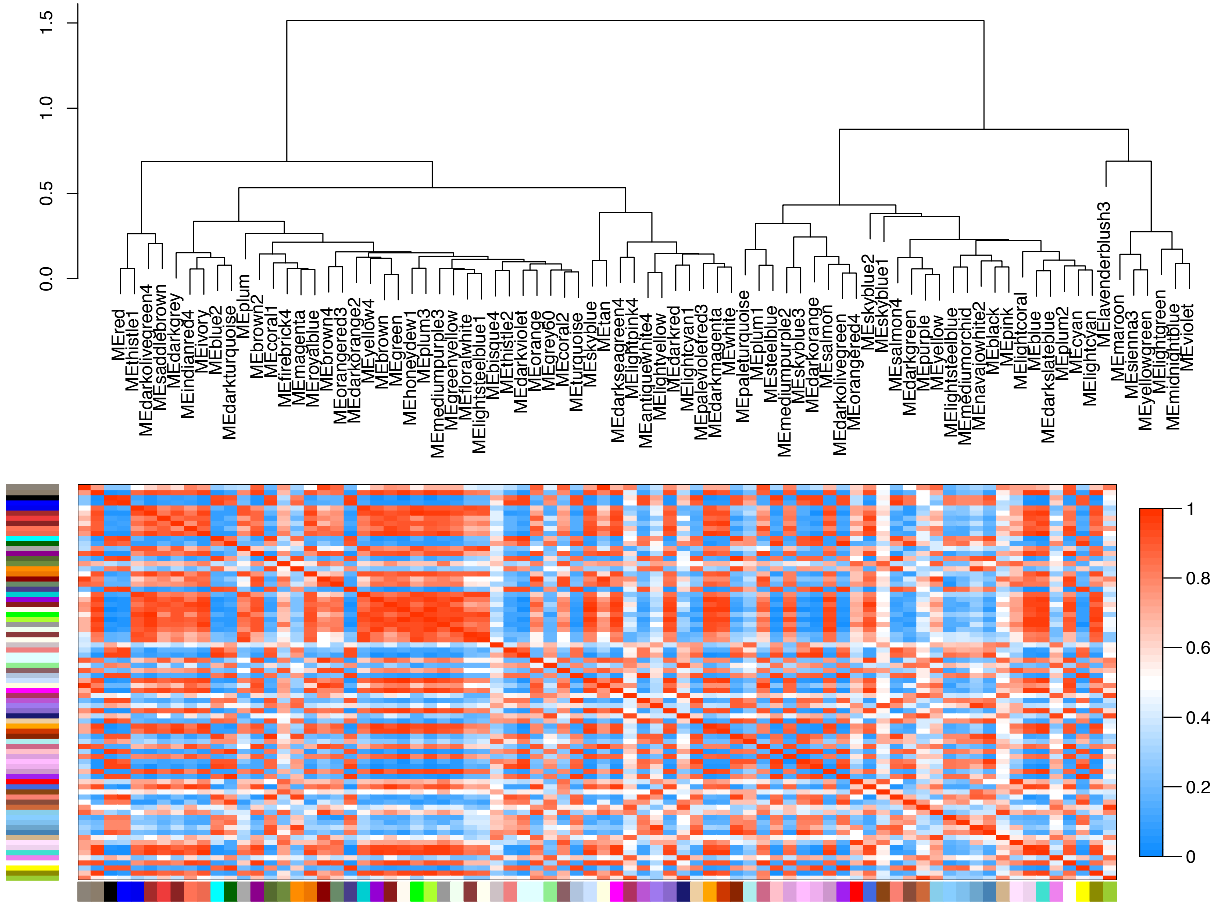

Figure S5: Module similarity by eigengene correlation. Analysis has been conducted on the filtered and normalized count matrix (27,027 unique genes) using WGCNA. The relationships of the initial 78 identified modules have been analyzed using the eigengenes as representative profiles and quantify module similarity by eigengene correlation. Note the clustering of the module eigengenes and their high similarity (above). Also the heatmap shows (below) shows the similarity of several modules based on the eigengene correlation.

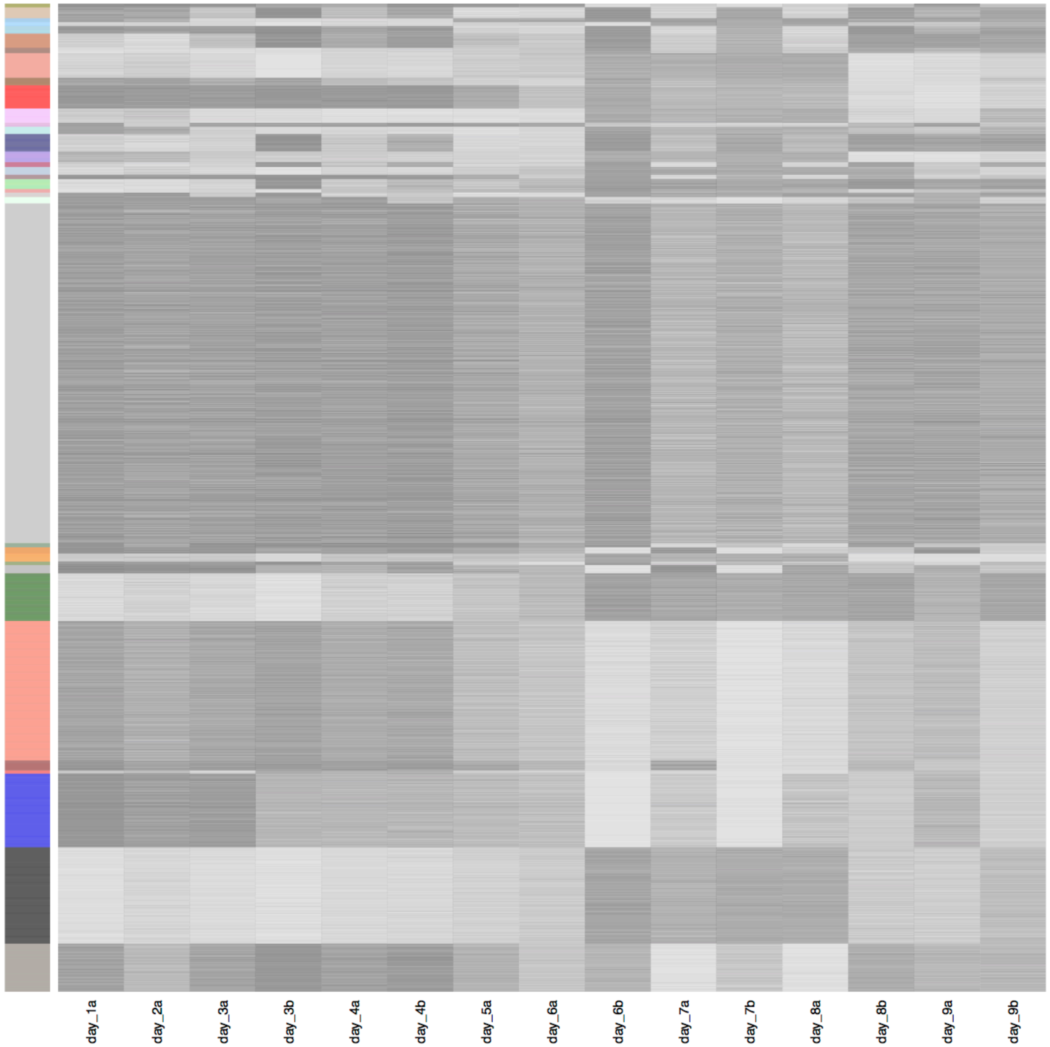

Figure S6: Gene expression across the embryonic development of *I. elegans* within the merged modules.

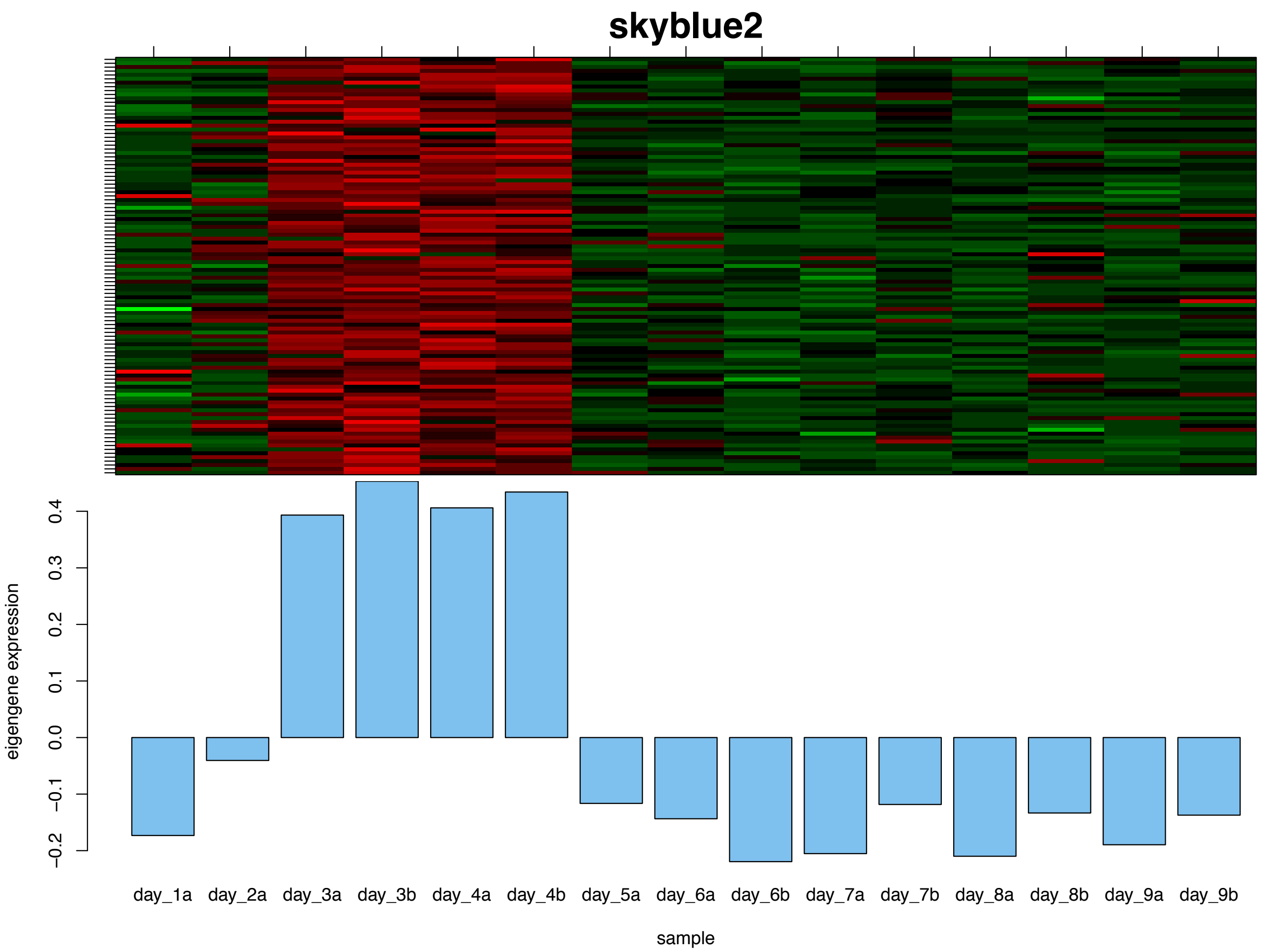

Figure S7: Heatmap and eigengene expression profiles of potential sex significant module. The y axis indicates the value of the module eigengene, the x axis the development and sample type. The skyblue2 module comprises 107 genes and a correlation coefficient of r = 0.98 with a Bonferroni adjusted nominal p-value 2.92×10^{-11}. Partition: 1-1-2-2-2-2-1-1-1-1-1-1-1-1-1 (1-2, male-female, female-male respectively).

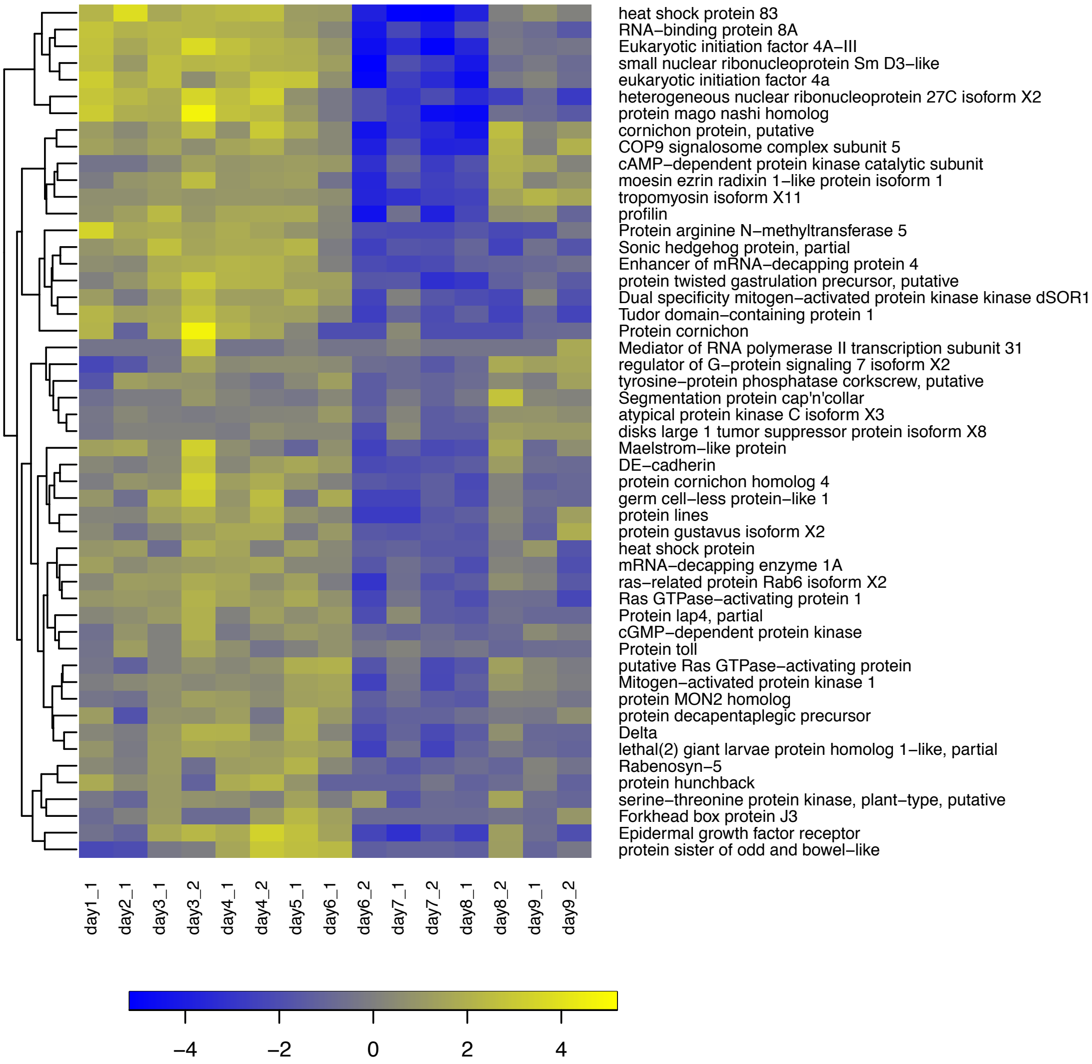

Figure S8: Expression dynamics of axis formation developmental process during *I. elegans* embryogenesis.

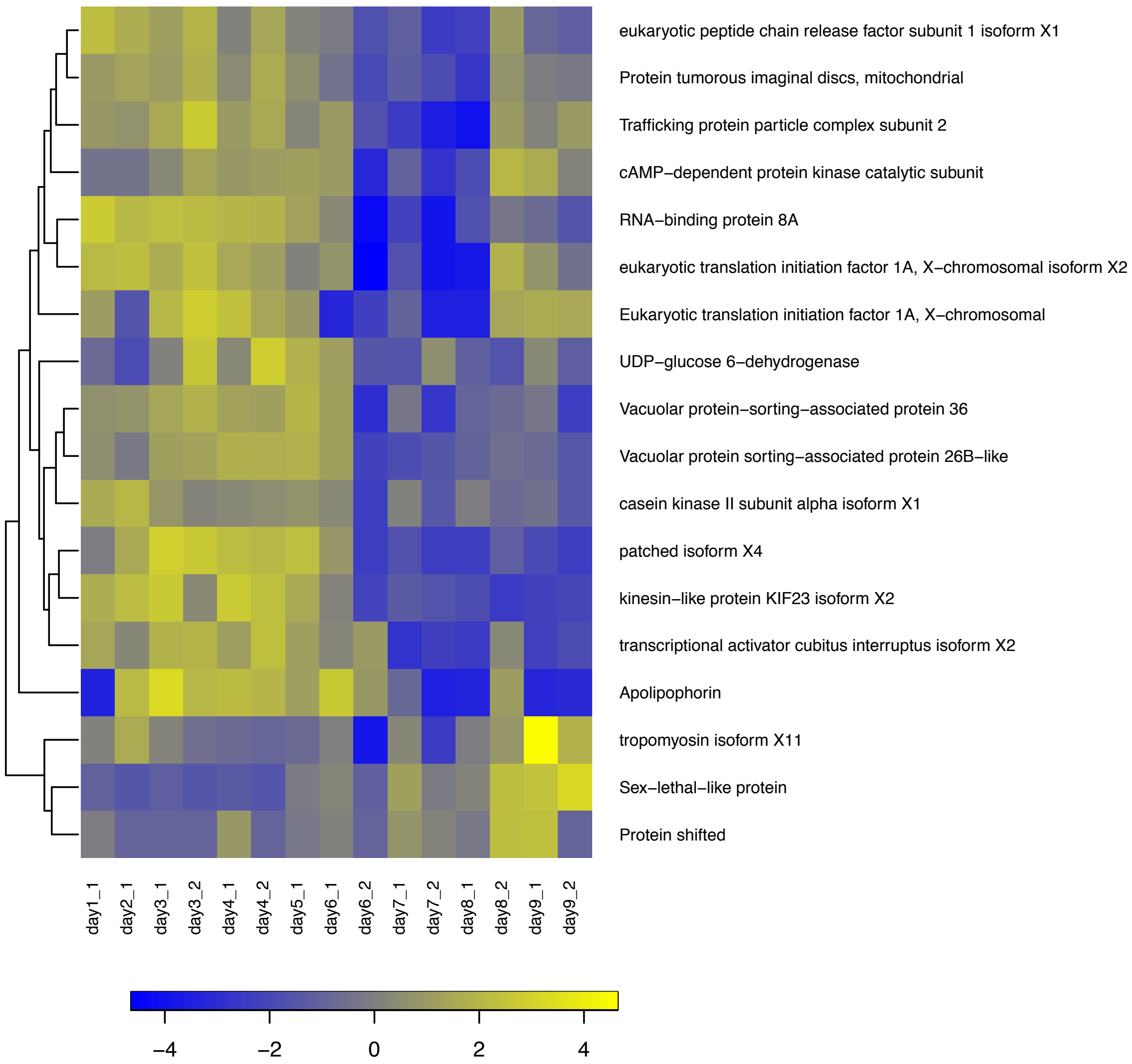

Figure S9: Expression dynamics of Hedgehog signaling pathway during *I. elegans* embryogenesis**.**

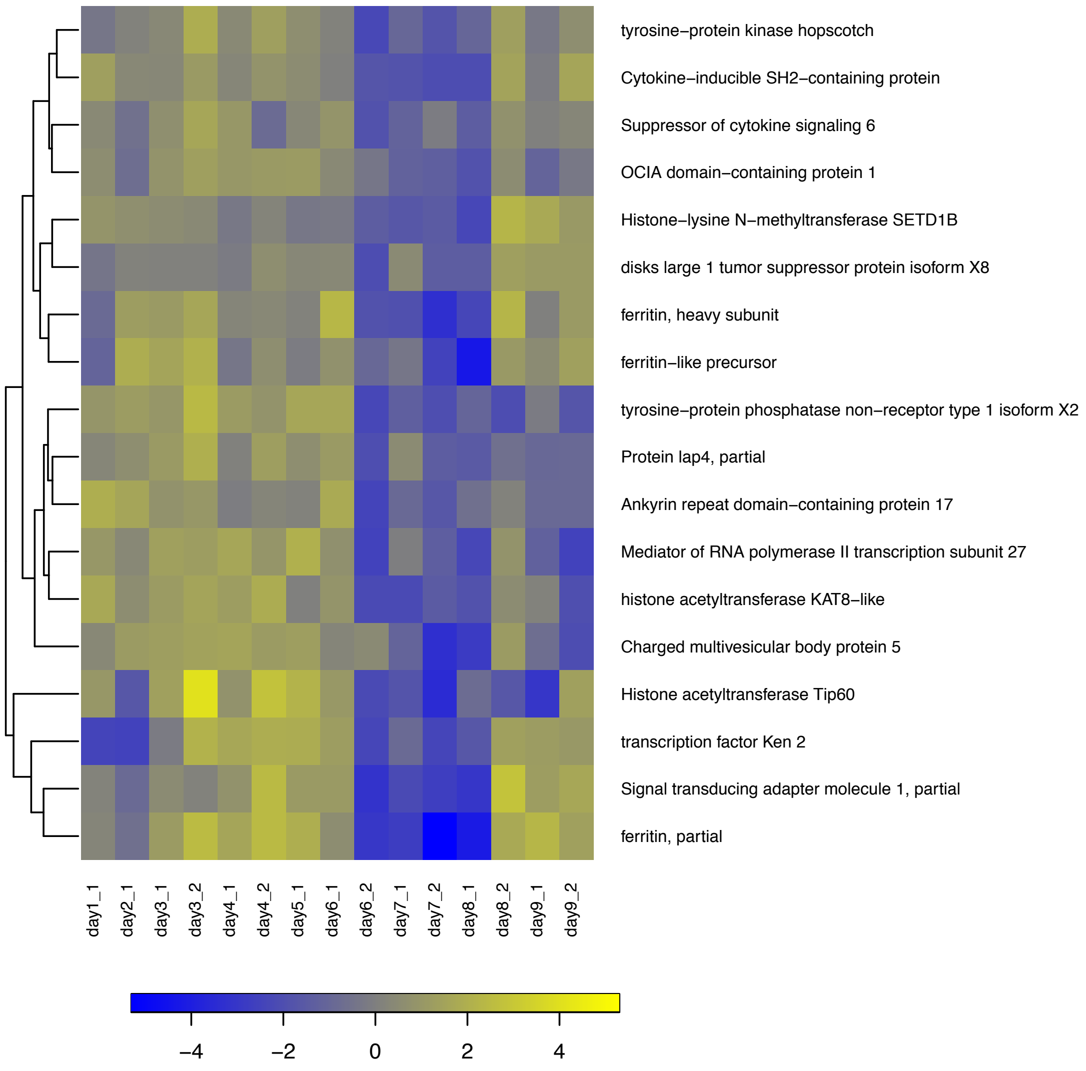

Figure S10: Expression dynamics of JAK-STAT signaling pathway during *I. elegans* embryogenesis**.**

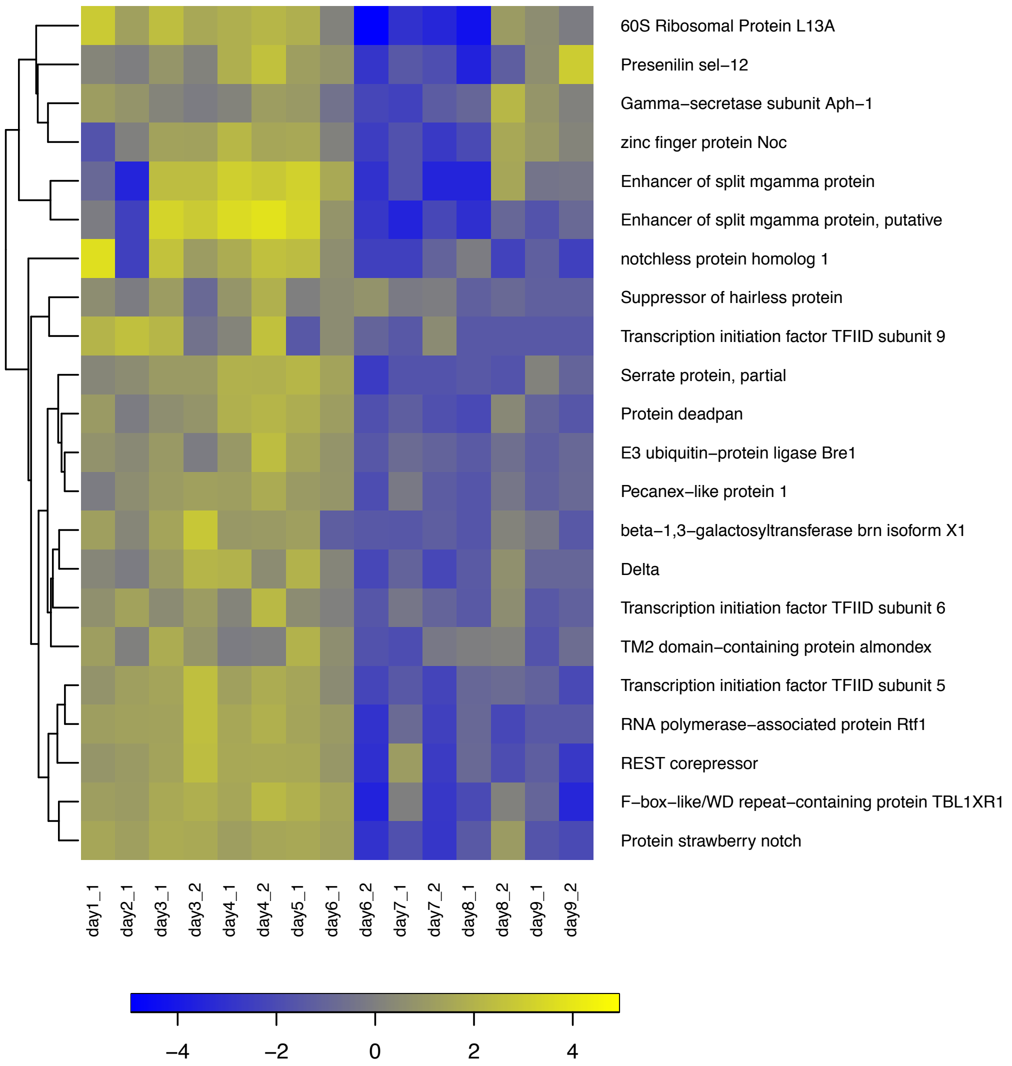

Figure S11: Expression dynamics of Notch signaling pathway during *I. elegans* embryogenesis.

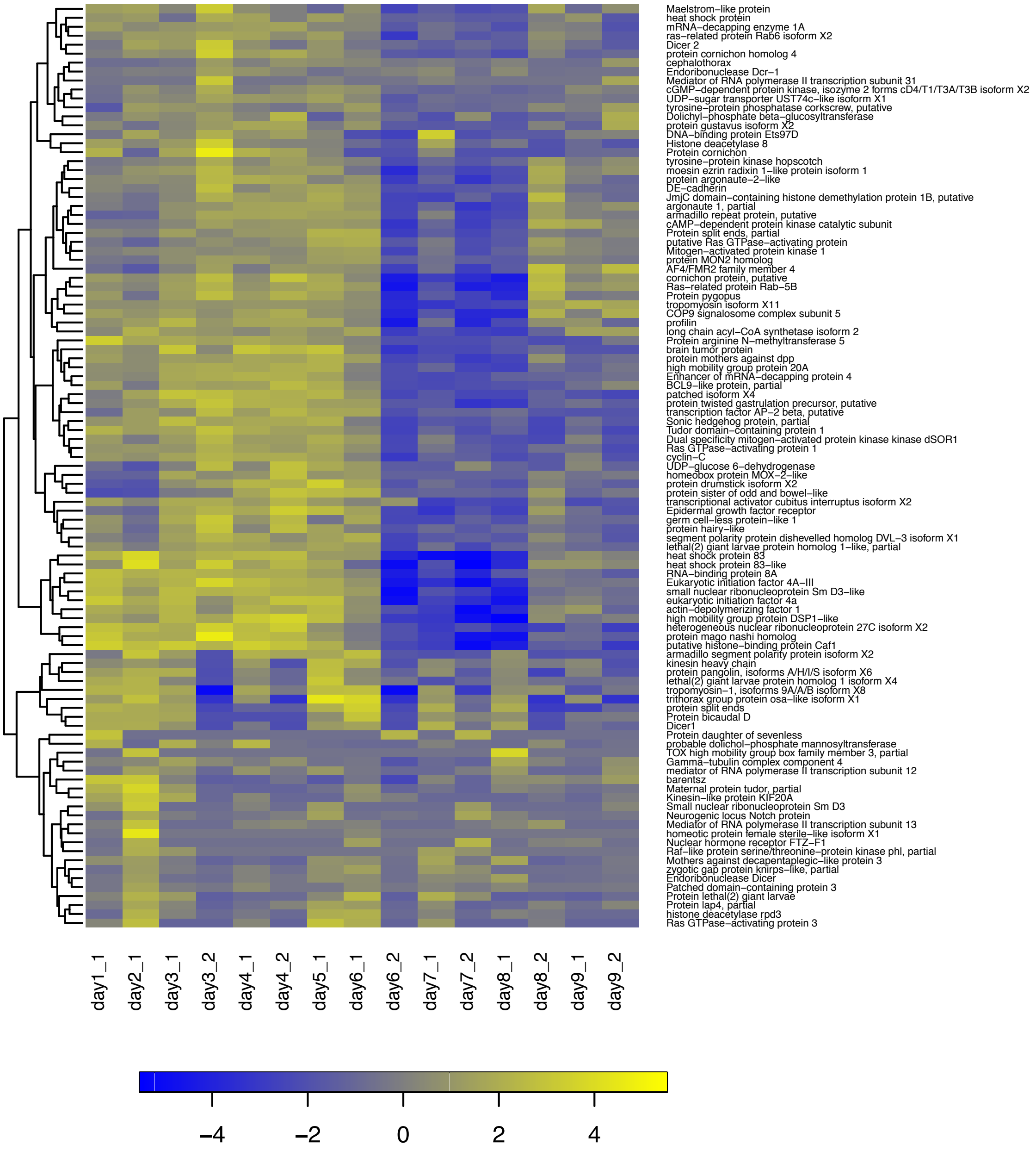

Figure S12: Expression dynamics of Segmentation development process during *I. elegans* embryogenesis**.**

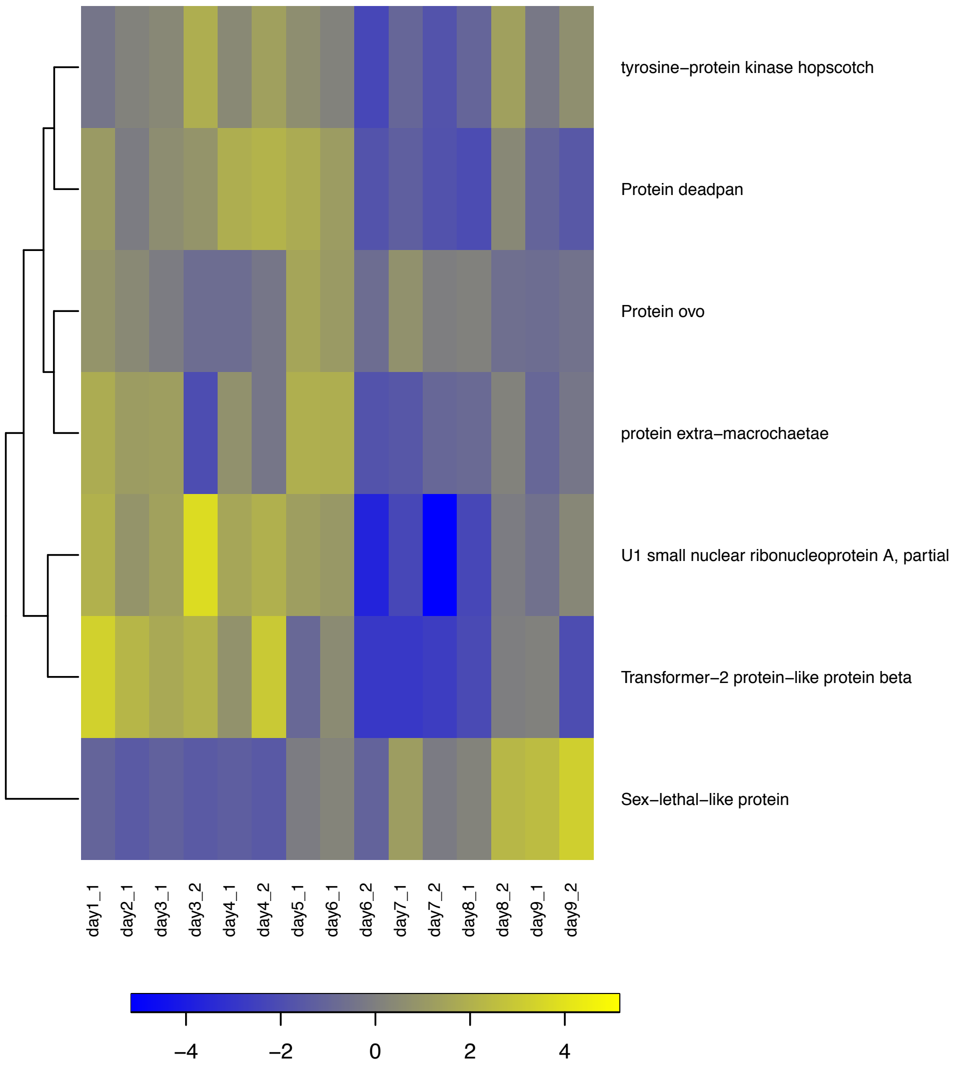

Figure S13: Expression dynamics of sex development process during *I. elegans* embryogenesis**.**

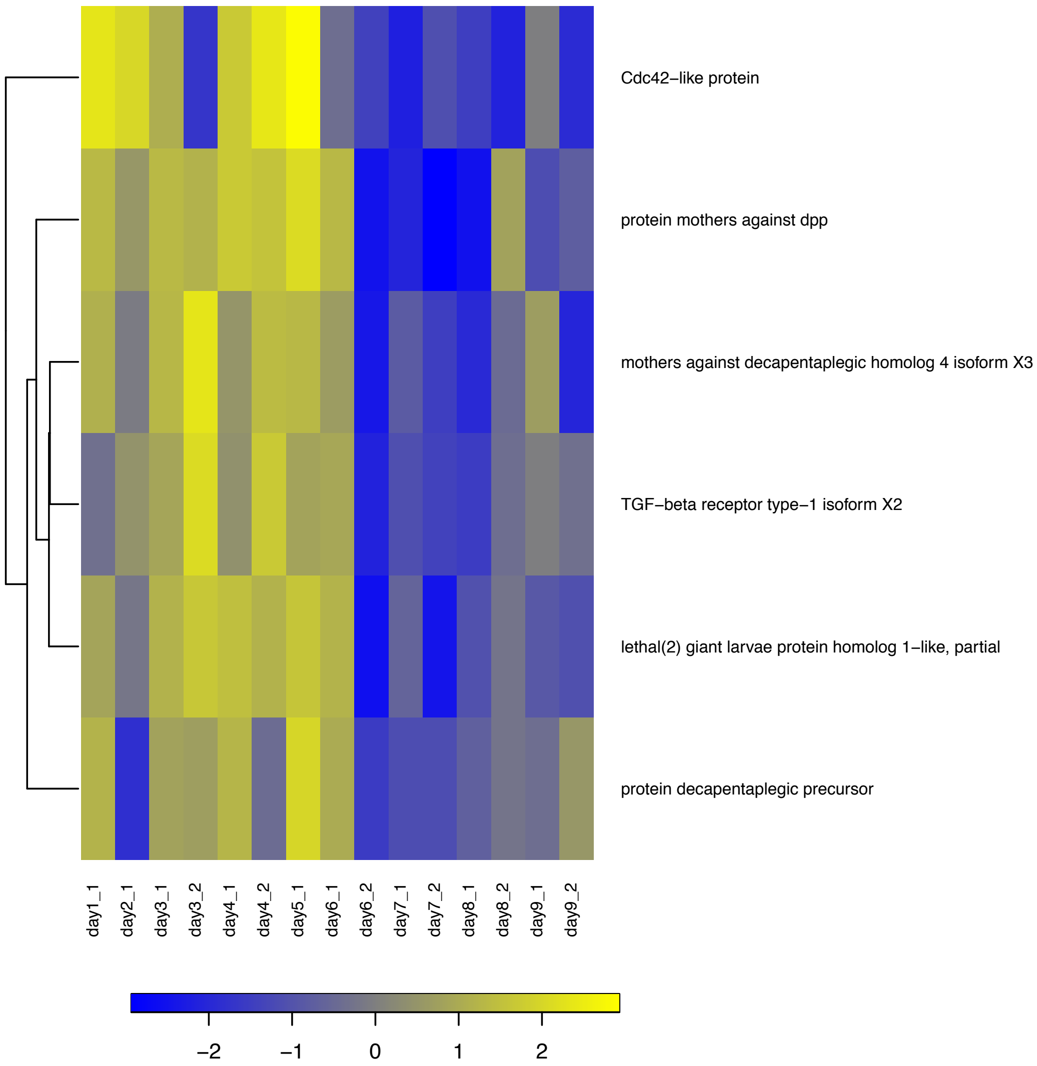

Figure S14: Expression dynamics of TGF-Beta signaling pathway during *I. elegans* embryogenesis**.**

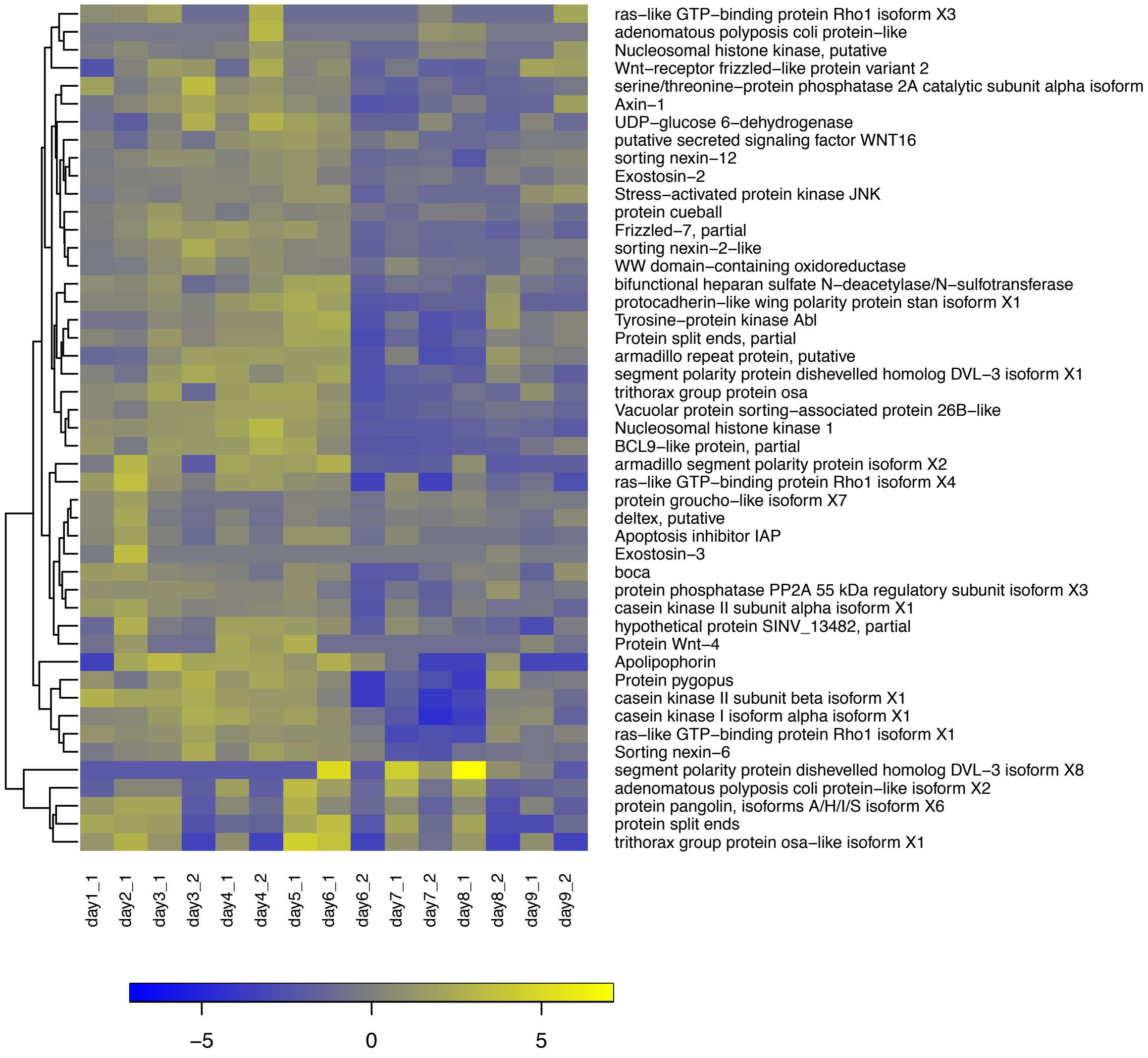

Figure S15: Expression dynamics of WNT signaling pathway during *I. elegans* embryogenesis**.**
